# Performance of reinforced concrete columns with reduced steel ratios strengthened by hybrid FRP/steel and high-strength concrete systems

**DOI:** 10.1038/s41598-026-49911-3

**Published:** 2026-05-25

**Authors:** Mohamed Ghalla, Ayah A. Alkhawaldeh, Alireza Bahrami, Rabeea W. Bazuhair, Yahya M. Bin Mahfouz, Galal Elsamak

**Affiliations:** 1 Civil Engineering Department, Faculty of Engineering, Kafrelsheikh University, Kafrelsheikh, Egypt; 2 Faculty of Engineering and Digital Technologies, University of Bradford, Bradford BD71DP, UK; 3 Department of Civil Engineering, Faculty of Engineering, Jerash University, Jerash, 26150 Jordan; 4 Department of Building Engineering, Energy Systems and Sustainability Science, Faculty of Engineering and Sustainable Development, University of Gävle, 801 76 Gävle, Sweden; 5 Civil Engineering Department, College of Engineering and Architecture, Umm Al-Qura University, Makkah, Saudi Arabia

**Keywords:** Fiber, High-strength concrete, Insufficient reinforcement, Reinforced concrete column, Steel ratio, Engineering, Materials science

## Abstract

Reinforced concrete (RC) columns with insufficient steel reinforcement present a significant structural concern. This issue is most commonly found in aging buildings, poorly constructed members, or structures designed before the adoption of modern codes. This study investigates the effectiveness of external hybrid strengthening techniques in enhancing the axial load-bearing capacities of such deficient columns. A series of rectangular RC column specimens with intentionally reduced longitudinal reinforcement ratios were tested under axial compression in the laboratory and simulated using ABAQUS software to evaluate structural performance. The specimens were strengthened using a combination of near-surface-mounted (NSM) steel or glass fiber-reinforced polymer bars with externally bonded glass fiber textile mesh covered with different types of high-strength concrete to form a hybrid strengthening solution. The influence of different strengthening configurations on the columns’ ultimate load, energy absorption, and failure modes was evaluated. The results show that the proposed hybrid systems improve the structural behavior of deficient RC columns. They also demonstrate that these systems significantly enhance the structural performance of deficient RC columns, achieving increases in the axial load-bearing capacity ranging from 11% to 78% compared with the deficient specimens. Additionally, the energy absorption capacity improved by up to 382%, highlighting the effectiveness of the combined NSM and external confinement techniques. Furthermore, these systems provide a practical solution for field engineers by helping them strengthen existing structures with inadequate internal reinforcement.

## Introduction

Insufficient steel reinforcement or degradation of reinforced concrete (RC) structures due to several external conditions (e.g., steel corrosion) are key structural engineering concerns^1–5^. In such cases, the reinforcing bars’ cross-sectional area is inadequate to withstand external loads or may be reduced by environmental conditions over their lifetime, which lowers their ductility, structural integrity, and load-bearing capacity. Considering the increased resistance of steel bars, traditional strengthening methods, such as patch repair and cathodic protection^6,7^, which are used to mitigate the loss of steel strength due to environmental conditions such as corrosion, do not compensate for the loss of reinforcement. In order to restore structural performance, external strengthening techniques such as steel jacketing, fiber-reinforced polymer (FRP) wrapping, and externally bonded reinforcement have been extensively researched^8^.

The concrete cover serves as the primary line of protection for steel reinforcing bars. A thicker layer of concrete improves this protective barrier by increasing resistance to environmental conditions. According to research, concrete columns with a thicker cover are significantly more resistant than those with a thinner layer^9^. The strength of the concrete itself is just as important in preventing losses in steel reinforcement as the thickness of the cover. Surface cracking and pitting, which are frequent routes for moisture and aggressive chemicals to reach the reinforcement, are less likely to occur with stronger concrete. Additionally, strengthening the concrete not only makes it more durable but also slightly raises the structural capacity of RC columns, increasing the ability to withstand axial loads^10,11^. In accordance with Bertolini et al.^12^, the main causes of reinforcement corrosion are carbonation and chloride penetration, which weaken the bond between steel and concrete and cause the concrete cover to delaminate and spall. The axial load-bearing capacity of columns can be considerably reduced by 10–20% if the diameter of the reinforcement is reduced, according to Almusallam^13^. Moreover, corroded reinforcement is brittle, which decreases RC columns’ ductility^14^.

In fact, there have been several methods to enhance the reinforcement ratio for deficient columns by implementing external strengthening methods. FRP composites, particularly carbon fiber-reinforced polymer (CFRP) and glass fiber-reinforced polymer (GFRP)^15,16^, are widely used for strengthening RC structures. According to previous research, externally bonded FRP wraps greatly enhance axial load-bearing capacity and confinement effectiveness^17^. For example, CFRP sheets wrapped around column surfaces can increase strength by up to 80%, depending on the wrapping layout. In addition to FRP composites, steel jacketing is a useful technique for covering deficient columns with steel plates or angles^18^. This approach improves both confinement and axial load-bearing capacity, making it ideal for restoring structural integrity in deficient or severely damaged columns. However, because columns are crucial elements in the structural skeleton, the strengthening process for these elements needs precise procedures to ensure long-term durability. Another efficient method is to use externally bonded reinforcement techniques such as near-surface-mounted (NSM) reinforcement and externally bonded steel plates^19^. NSM-FRP bars, in particular, have demonstrated significant potential for increasing the flexural and shear strengths of RC elements. This approach provides a long-lasting and non-intrusive solution, which makes it ideal for applications that require minimum structural changes. Moreover, the use of ultra-high-performance concrete (UHPC), engineered cementitious composites (ECCs), and fiber-reinforced cementitious mortar has emerged as viable strengthening materials due to their excellent mechanical strength and durability^20^. Previous studies found that UHPC overlays improve the load-bearing capability of deficient RC members while offering superior environmental resistance. UHPC confinement techniques provide advantages over standard jacketing approaches, especially in extreme environmental conditions^21,22^.

To further optimize structural performance, a combination of strengthening approaches has been proposed. Hybrid techniques, such as combining FRP wrapping with NSM reinforcement or integrating steel jacketing with FRP confinement, have outperformed single-method applications^23^. Experimental investigations indicate that these combined systems are beneficial in situations of inadequate reinforcement provided in structural members. These strengthening methods can improve the strength, capacity, and overall durability of deficient RC members.

To better understand the structural behavior of RC elements such as beams, columns, and beam-column joints strengthened using various techniques, finite element modeling (FEM) has become a widely used and reliable tool. In accordance with Karimipour and Edalati^24^, FEM provides accurate predictions of how RC members perform, including those that have been strengthened. In addition to simulation studies, experimental research has confirmed the effectiveness of several strengthening methods, especially those involving FRPs and UHPC, in notably improving structural capacity^25^.

A review of the existing literature reveals a significant knowledge gap regarding the combined use of fiber-reinforced technology and UHPC for the strengthening of RC columns. Although various strengthening techniques have been investigated, the integration of fiber systems with UHPC has received limited attention, emphasizing the need for more extensive research in this area. In response to this gap, the present study investigates a hybrid strengthening strategy that combines NSM steel or GFRP bars with externally bonded glass fiber textile mesh (GFTM) covered with different types of high-strength concrete for the rehabilitation of RC columns with inadequate steel reinforcement. The effectiveness and performance of this hybrid strengthening technique have been evaluated in this study by conducting experimental testing and developing a nonlinear finite element analysis (NLFEA) to validate the experimental findings.

Despite the extensive use of various strengthening techniques for RC columns, limited attention has been given to hybrid systems that combine NSM reinforcement with fiber-based confinement integrated within high-strength concrete overlays. In addition, existing studies often focus on individual strengthening methods without fully addressing their combined structural efficiency. Therefore, there is a need for a more integrated approach that enhances both load-bearing capacity and ductility in columns with insufficient internal reinforcement. This study aims to address this gap by investigating a hybrid strengthening system that combines NSM bars with GFTM and high-strength concrete jackets.

## Research objectives

**Fig. 1:**
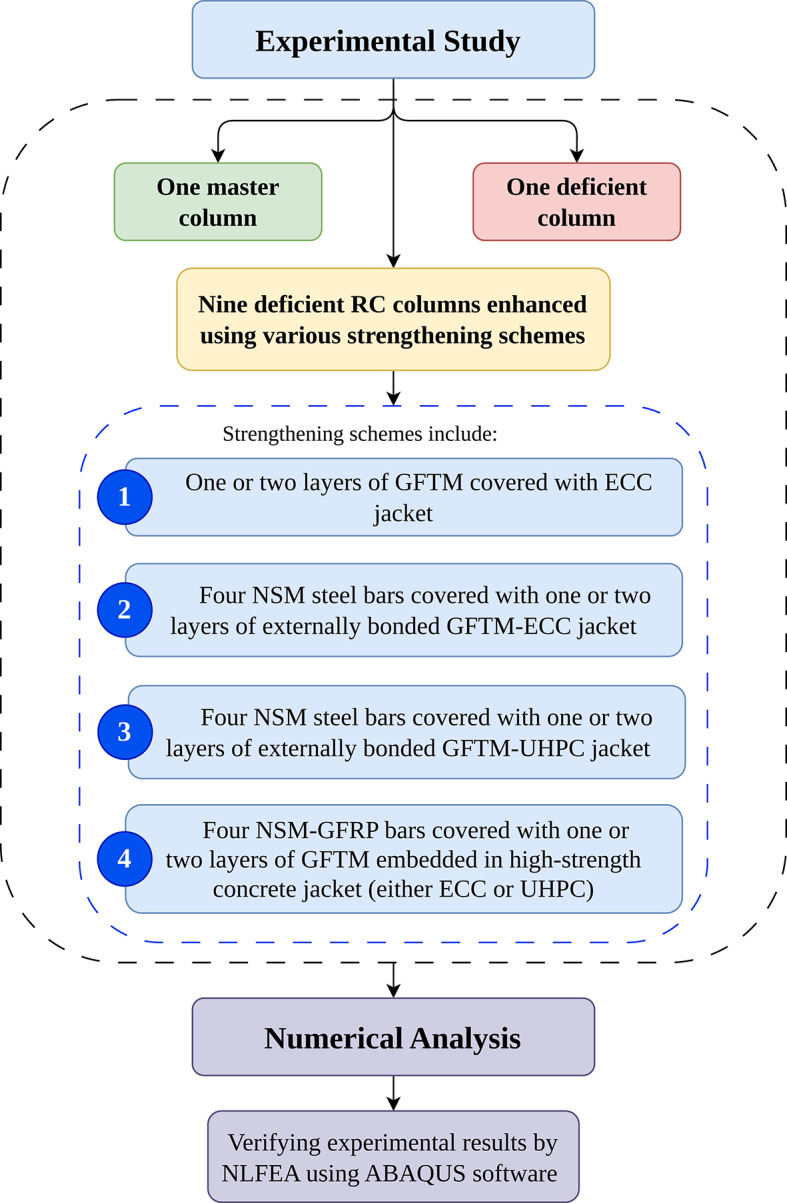
Applied test matrix.

This study aims to assess the efficacy of external strengthening methods in improving the axial load-bearing capacity of RC columns with insufficient internal reinforcement. To address these deficiencies, several strengthening strategies were implemented, including hybrid systems that integrate NSM steel or GFRP bars with externally bonded GFTM. These reinforcement elements were embedded beneath high-strength concrete overlays made of either ECC or UHPC. Moreover, the strengthening of columns using an external GFTM-ECC jacket was also investigated as a standalone retrofitting system. An NLFEA was further applied, verifying the experimental findings. The testing program included the preparation of a reference column, designated as the master column (i.e., the reference column), along with ten deficient columns. One of the deficient columns was left without strengthening as a control specimen, while the other nine columns were strengthened using different techniques. The applied test matrix is illustrated in Fig. 1.

## Testing methodology and guidelines

This study evaluated a hybrid strengthening strategy used for strengthening RC columns with insufficient reinforcement through experimental testing and NLFEA modeling. Eleven RC columns were cast using normal concrete (NC) and conventional steel reinforcement. These columns were strengthened externally employing various methods: (1) GFTM covered with different types of high-strength concrete and (2) a hybrid system combining NSM steel or GFRP bars with externally bonded GFTM, also covered with a high-strength concrete jacket. The performance of the strengthened columns was compared with that of the reference columns in terms of failure modes, energy absorption, displacement, and maximum axial load-bearing capacity. The following sections present the column fabrication process, reinforcement configurations, materials used, preparation techniques, test setup, and instrumentation.

### Columns details

**Fig. 2:**
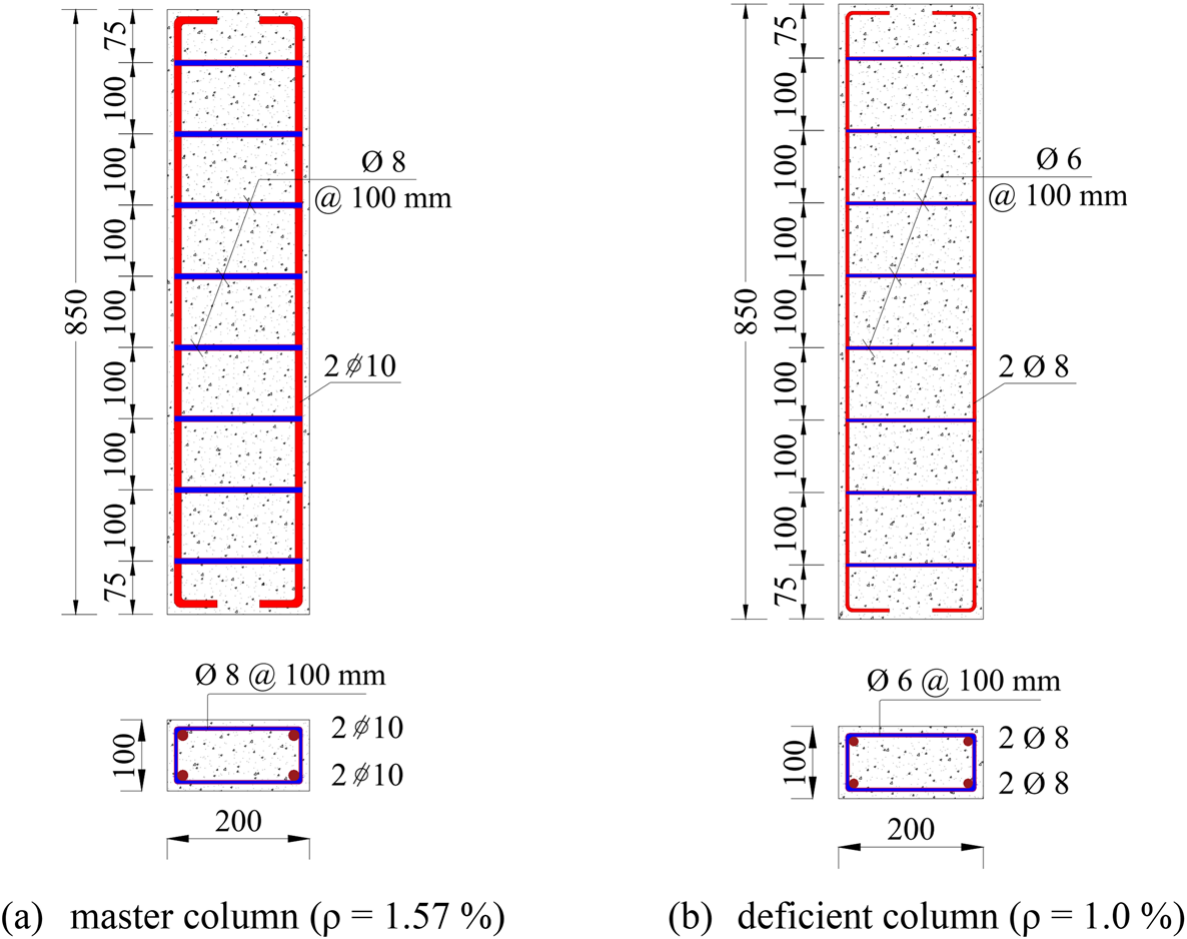
Physical properties and reinforcement details of tested columns (units: mm).

In this study, one column was designated as the control or master column, reinforced with 4φ10 mm longitudinal bars, having a reinforcement ratio (ρ) of 1.57%, and confined with φ8 mm ties spaced at 100 mm center-to-center. The remaining ten columns were designed with reduced reinforcement, reinforced with 4φ8 mm longitudinal bars, with a reinforcement ratio of 1.00%, and confined with φ6 mm ties at 100 mm spacing. This reduction in reinforcement was intended to simulate insufficient steel reinforcement area, representing deficient columns. External strengthening techniques were applied to these deficient columns. All columns had identical geometric dimensions, with a length of 850 mm and a cross-section of 200 mm by 100 mm. The geometric configurations and reinforcement details of the tested columns are displayed in Fig. 2.

The deficient columns were strengthened externally using four distinct reinforcement schemes: Scheme 1: One column was reinforced with a single layer of GFTM covered with ECC mortar, while another column was strengthened with two layers of GFTM-ECC. Scheme 2: Two columns were strengthened with four NSM steel bars in combination with either one or two layers of GFTM-ECC. Scheme 3: Two columns were reinforced with four NSM steel bars and either one or two layers of GFTM, covered with UHPC, forming a strengthening system known as ultra-high-performance fiber-reinforced concrete (UHPFRC) jacket. Scheme 4: Three columns were strengthened with four NSM-GFRP bars, combined with either one or two layers of GFTM-ECC jacket or one layer of UHPFRC jacket. The details of these strengthening schemes are listed in Table 1. All columns were constructed using NC with the properties indicated in Table 2.

The selection of test variables was based on simulating practical cases of deficient RC columns with reduced steel ratios and evaluating commonly used strengthening techniques. The variation in reinforcement ratios was adopted to represent typical deficiencies in existing structures, while different strengthening configurations were selected to assess the individual and combined effects of NSM reinforcement and external confinement. Additionally, ECC and UHPC were utilized to investigate the influence of high-strength materials on the overall structural performance.

**Fig. 3:**
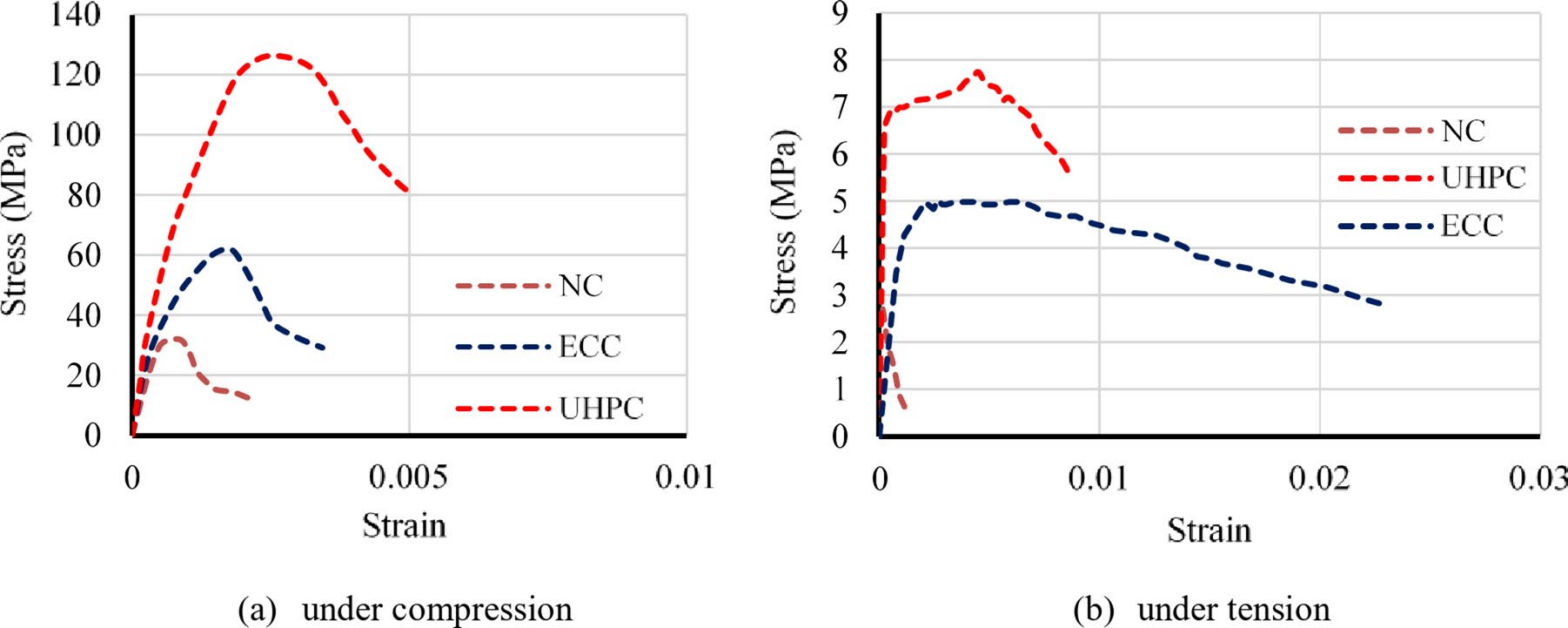
Stress–strain relationship for concrete.

**Table 1:** Details of tested columns (IDs, parameters, strengthening schemes).

Group	Specimens’ ID	Studied parameter	Number of used GFTM	Concrete type for confining jacket	Volume ratio of vertical bars used for column strengthening
G1	C0	Master column	–	–	–
	DC	Deficient column	–	–	–
G2	DC	GFTMs covered with ECC jacket	–	–	–
	DC-G1-E		One	ECC	–
	DC-G2-E		Two	ECC	–
G3	DC	Four NSM steel bars covered with externally bonded GFTM-ECC jacket	–	–	–
	DC-0.57-G1-E		One	ECC	0.57%
	DC-0.57-G2-E		Two	ECC	0.57%
G4	DC	Four NSM steel bars covered with externally bonded UHPFRC jacket	–	–	–
	DC-0.57-G1-U		One	UHPFRC	0.57%
	DC-0.57-G2-U		Two	UHPFRC	0.57%
G5	DC	Four NSM-GFRP bars covered with high-strength concrete jacket (either ECC or UHPC)	–	–	–
	DC-1.57G-G1-E		One	ECC	1.57%
	DC-1.57G-G2-E		Two	ECC	1.57%
	DC-1.57G-G1-U		One	UHPFRC	1.57%

### Materials properties

#### NC, ECC mortar, and UHPC

A high-quality NC mixture was produced using ordinary Portland cement, fine aggregate, coarse aggregate with a maximum particle size of 10 to 20 mm, and water. The mix was designed according to the standard procedures outlined in ACI 211.1–91. The RC columns were cast for experimental study using this concrete. Also, an ECC mortar was made with ordinary Portland cement, fine aggregate, fly ash, silica fume, chopped polyvinyl alcohol (PVA) and steel fibers (2% of the concrete volume), water, and a high-range water reducer (HRWR). Furthermore, a UHPC mix was produced using ordinary Portland cement, fine aggregate, silica fume, chopped PVA and steel fibers (2% of the concrete volume), water, and a HRWR.

The PVA fibers used in the mix measured 12 mm in length and had a tensile strength of 1200 MPa, an elastic modulus of 40 GPa, and an elongation of 5.0%. The steel fibers used in the mixes had a hooked-end deformed shape, which enhances their bond with the cement matrix and improves the overall mechanical performance. The steel fibers had a tensile strength of 1172 MPa, which allows them to resist cracking and increase the toughness of concrete. Their elastic modulus was 202 GPa, and their density was 7850 kg/m³. This density ensures good dispersion within the concrete mix and offers enhanced durability and structural integrity. The RC columns were reinforced with GFTM, which was integrated into ECC or UHPC mortar.

Standard cylinders of 200 mm in length and 100 mm in diameter were cast from various batches of concrete and tested in compliance with ASTM C39 standards to determine the concrete’s 28-day compressive strength. Table 2 provides a summary of the mix proportions, compressive strengths (f'c), and Poisson’s ratios. In addition, uniaxial tensile tests were performed on dog-bone-shaped specimens to assess the tensile stress-strain behavior of each concrete type. Figure 3 shows the tensile stress-strain curves collected from these laboratory tests.

**Table 2:** Concretes mix design details and compressive strengths.

Concrete	Cement (kg/m^3^)	Fine aggregate (kg/m^3^)	Coarse aggregate (kg/m^3^)	Fly ash (kg/m^3^)	Silica fume	Water/binder	PVA/steel fiber (%) in volume	HRWR (kg/m^3^)	f'_c_ (MPa)
NC	350	700	1150	–	–	0.42	–	–	30
ECC	560	442	–	610	235	0.25	2.00	14.6	62
UHPC	900	1005	–	–	220	0.22	2.00	40.3	126

#### Reinforcements

The master column was reinforced with Grade 60 steel for the main reinforcement (10 mm diameter) and Grade 40 steel for the shear reinforcement (8 mm diameter). In contrast, the deficient columns were reinforced entirely with Grade 40 steel, including 8 mm diameter longitudinal bars and 6 mm diameter ties. All steel reinforcement conforms to ASTM A615/A615M standards, ensuring compliance with the required mechanical properties for RC structures.

**Fig. 4:**
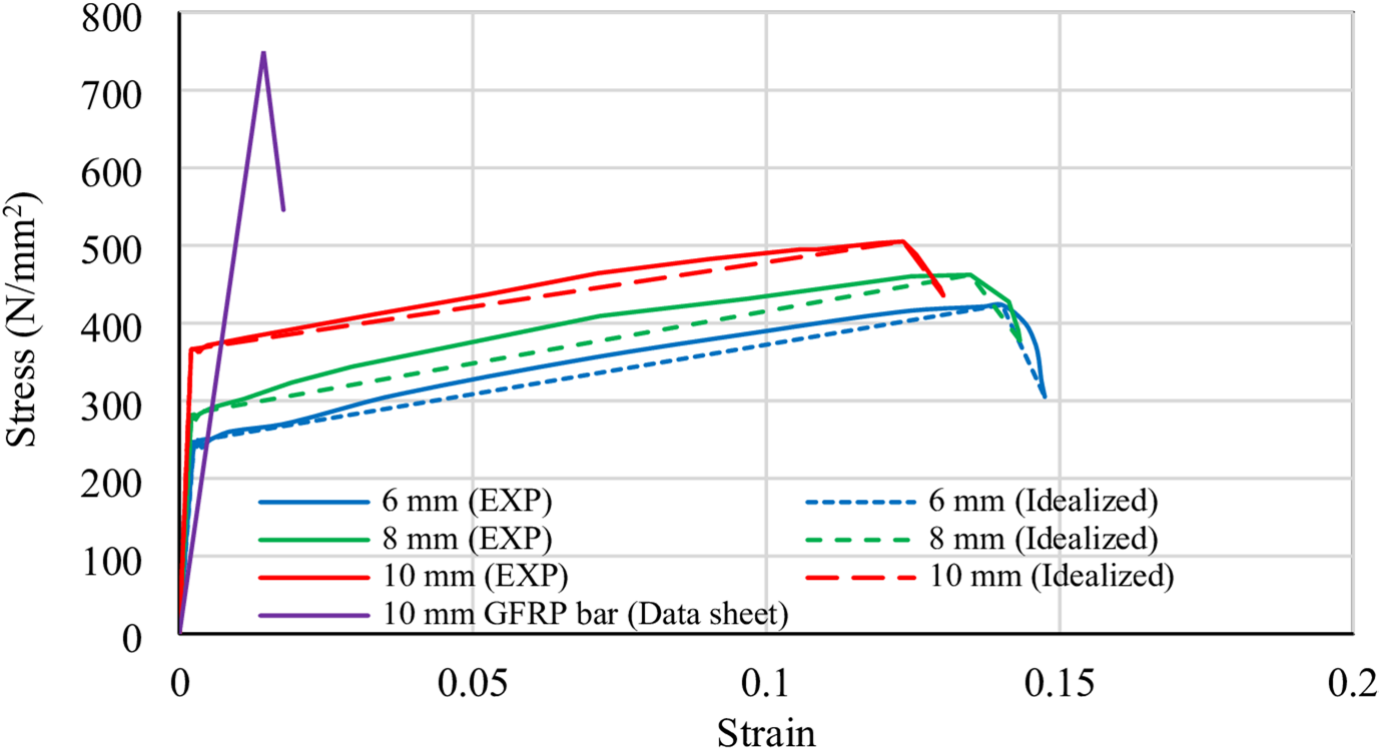
Stress–strain relationship for reinforcing bars.

The mechanical properties of the steel bars were determined through tensile tests in accordance with ASTM A370. For the GFRP bars, mechanical properties were based on the manufacturer’s data sheet. Table 3 summarizes key properties, including strength, strain, elastic modulus, and Poisson’s ratio. The stress-strain behavior of steel and GFRP reinforcement is depicted in Fig. 4, which provides a clear comparison of their mechanical behavior.

**Table 3:** Mechanical properties of structural steel bars.

Material	Yield strength		Ultimate strength		Elastic modulus (GPa)	Poisson’s ratio
	σ_y_ (MPa)	ϵ_y_ (%)	σ_u_ (MPa)	ϵ_u_ (%)		
Steel bar (6 mm diameter)	247	1.33	423	14.01	185	0.30
Steel bar (8 mm diameter)	283	1.47	462	13.47	192	0.30
Steel bar (10 mm diameter)	366	1.78	505	12.33	205	0.30
GFRP bars	–	–	750	1.45	50	0.27

#### GFTM

In this study, a GFTM, specifically Sika Fiber Mesh 1000, was used as an external non-metallic reinforcement to improve the axial load-bearing capacity of RC columns. The mesh is made of two-dimensional glass fiber strands, with fibers spaced 5 mm apart in both the horizontal and vertical directions. Each strand forming the mesh was 0.12 mm thick and 0.85 mm wide. According to the specifications provided by the manufacturer, GFTM had a weight of 74 g/m², an elastic modulus of 70 GPa, and an ultimate tensile strength of 900 MPa, with a failure elongation of approximately 1.2%.

### Casting and strengthening procedures

**Fig. 5:**
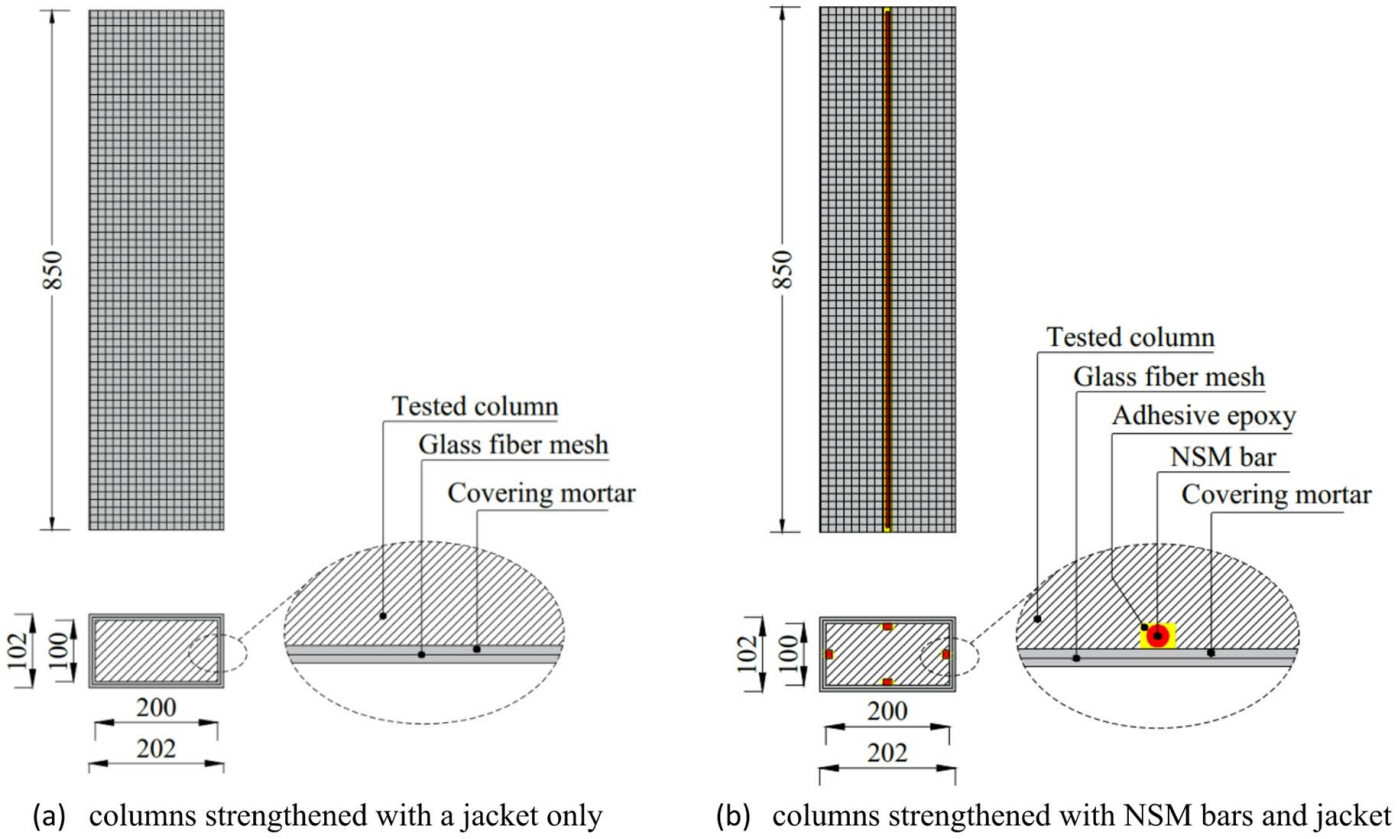
Strengthening schemes (units: mm).

All RC columns in this study were constructed using standard concrete and structural steel reinforcement, as described in detail in the preceding sections. Following a standard 28-day curing period, the columns were ready for the application of the strengthening schemes. The different strengthening configurations used in the study are illustrated in Fig. 5.

**Fig. 6:**
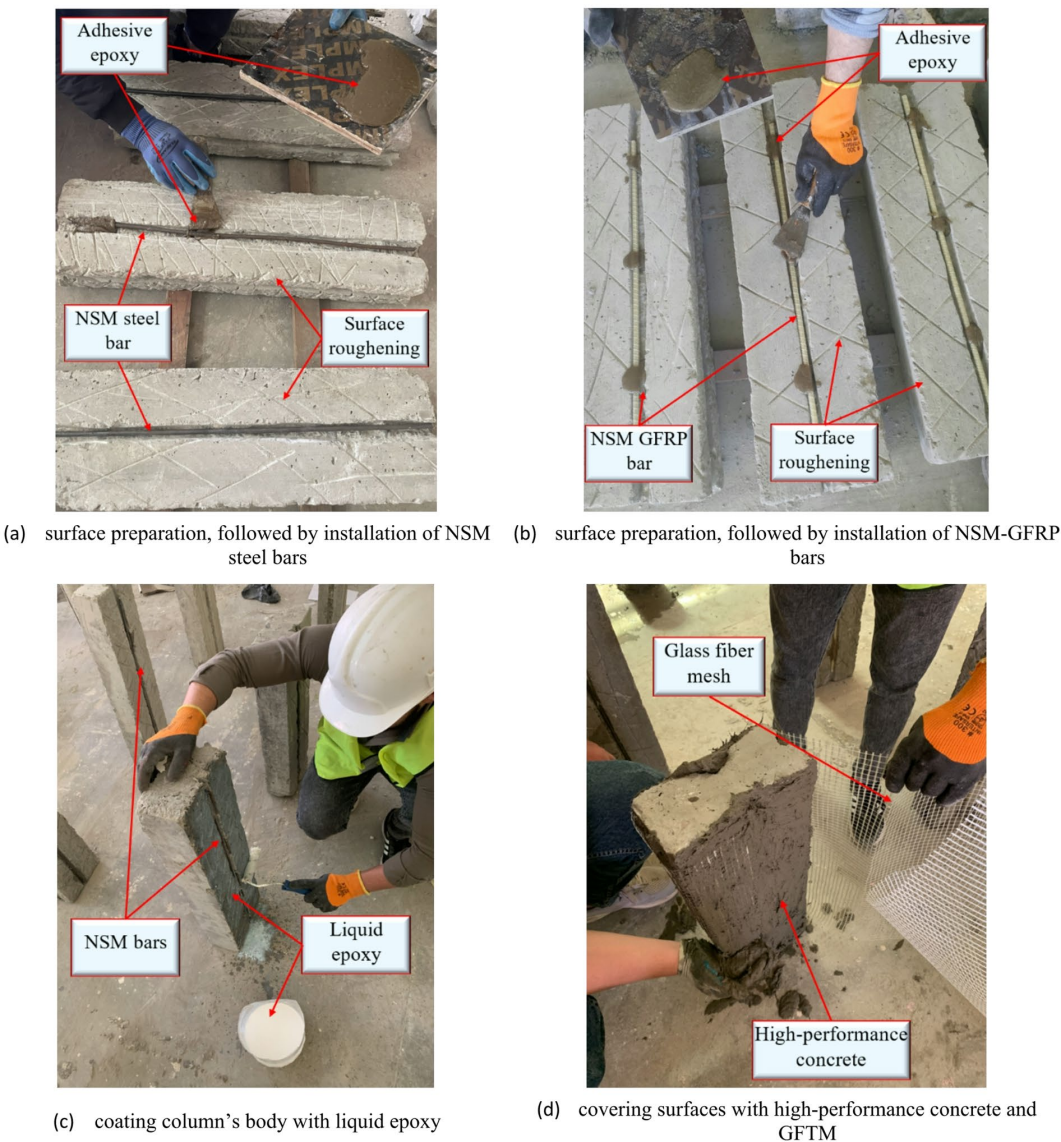
Strengthening preparation.

To ensure strong and durable bonding, a systematic strengthening procedure was adopted. The concrete surfaces were first cleaned and roughened to improve adhesion between the existing substrate and the applied strengthening materials. For specimens strengthened using the NSM technique, 25 mm wide and 15 mm deep grooves were cut into the concrete surface to install the reinforcement. The external reinforcement bars (i.e., either steel or GFRP bars) were then placed inside these grooves, and epoxy adhesive was applied at several points between the bars and the grooves to optimize bond strength. Next, a thin layer of liquid epoxy was applied to the entire column surface, followed by the application of high-strength concrete, which was either ECC or UHPC. Subsequently, GFTM was placed over the surface, and another layer of high-strength concrete was applied. This layering process was repeated based on the required number of GFTM layers. Proper surface preparation, which included cleaning and roughening, played a crucial role in improving the bond strength between the existing concrete substrate and the GFTM-ECC or UHPFRC layers. Once the mesh layers were in place, a final layer of high-strength concrete (either ECC or UHPC) was applied to provide a smooth, durable finish. The complete strengthening procedure carried out in the lab is presented in Fig. 6. After strengthening, all RC columns were cured at room temperature for an additional 28 days before undergoing mechanical testing.

### Instrumentation, loading configuration, and test setup

**Fig. 7:**
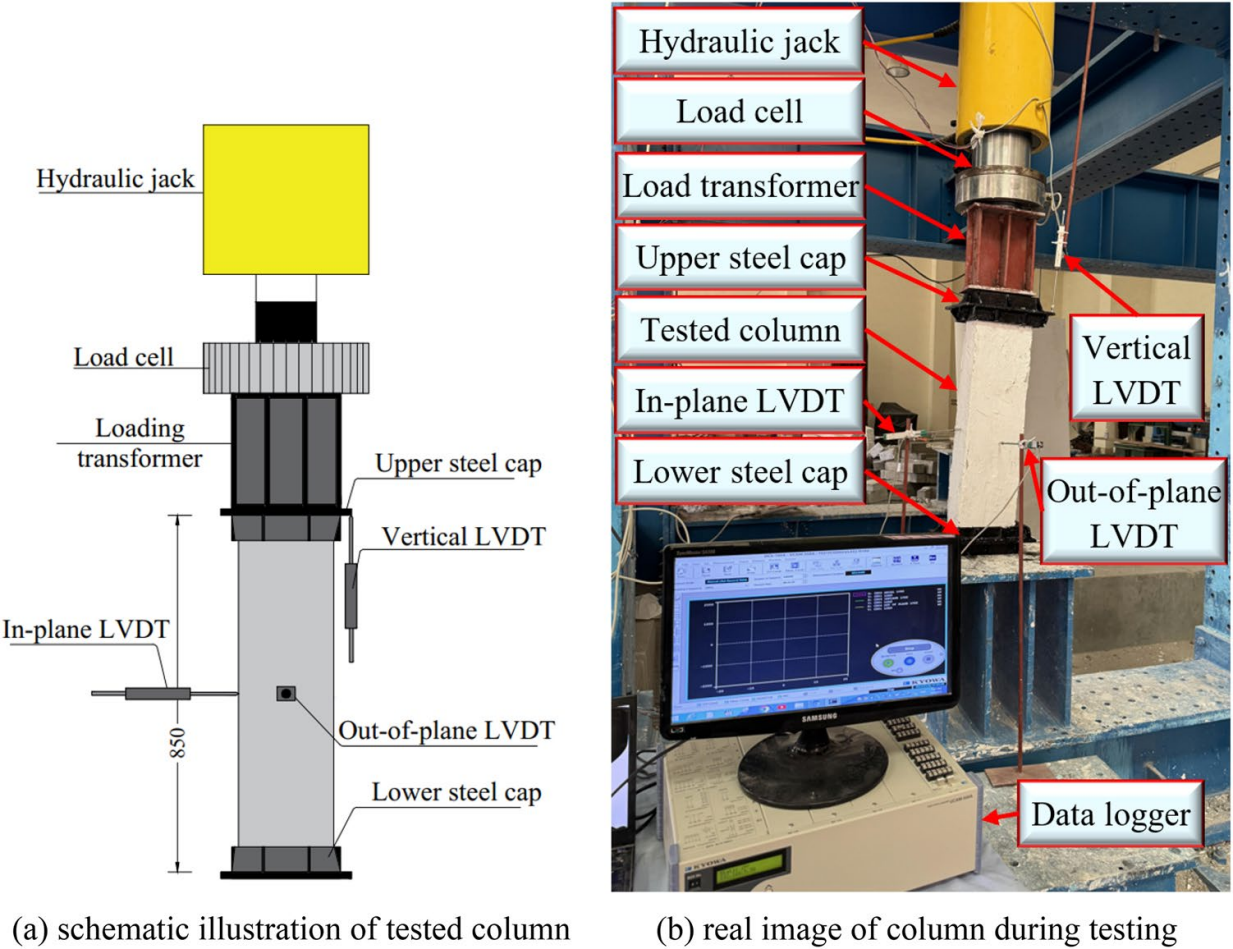
Test setup and details of instrumentation (unit: mm).

All columns, including the reference and strengthened specimens, in this study were tested under axial loading, which was performed using a monotonic load-controlled procedure. A 1000 kN capacity load cell was utilized to measure the applied axial force with high accuracy, and a hydraulic jack coupled to the load cell, which was connected to a loading transformer, was used to progressively increase the applied load. Load and displacement were recorded at each loading increment using a data logger. All specimens were kept under identical boundary conditions to ensure testing uniformity. Linear variable differential transducers (LVDTs) were installed at the mid-height of each column (on two faces of the column), with one vertical LVDT employed to measure axial displacement. The experimental setup shown in Fig. 7 consists of a loading device, a restraint system, and steel caps at both ends of the columns to ensure the uniformity of load distribution and to prevent early localized stress concentrations.

## Results and discussion

### Failure modes

**Fig. 8:**
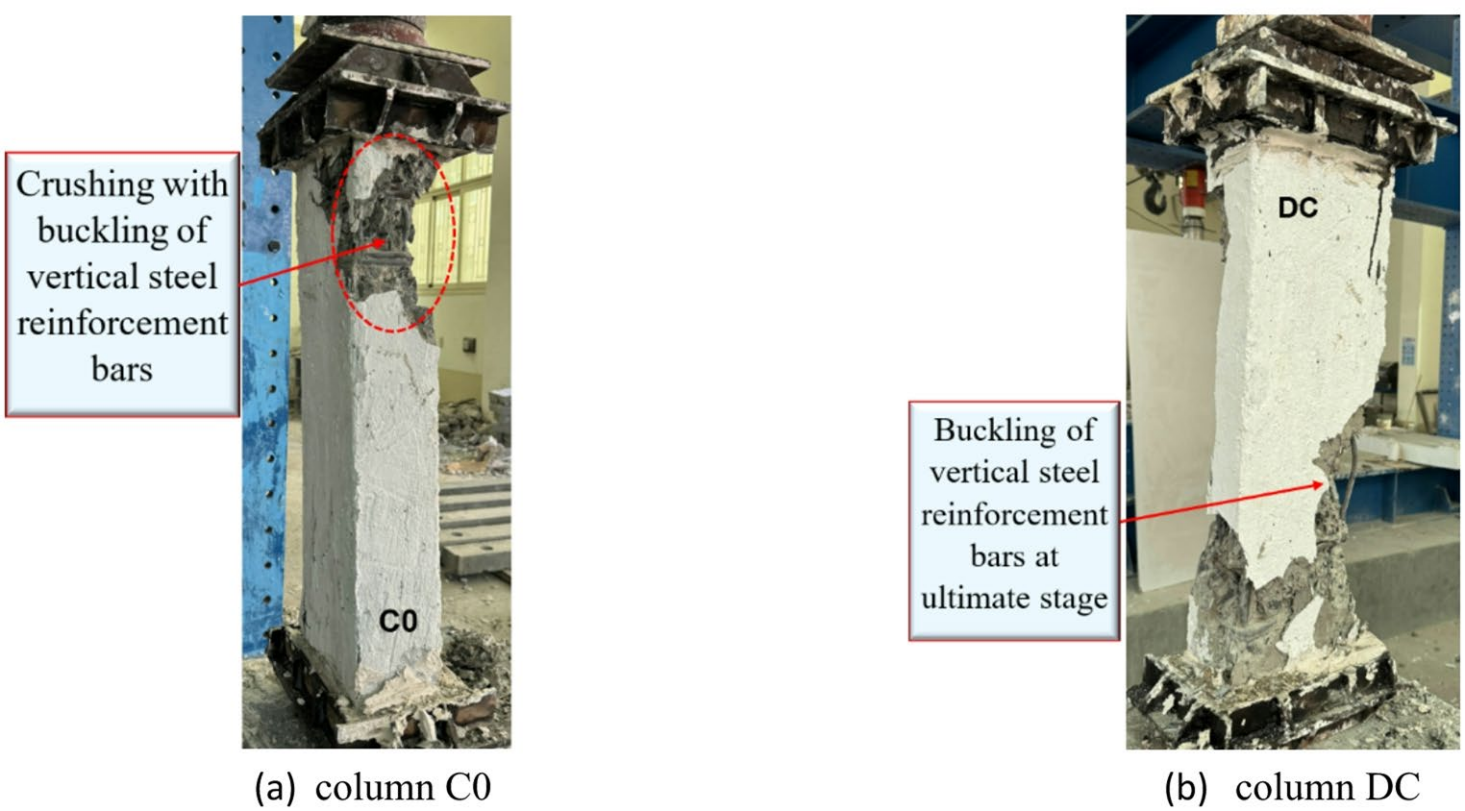
Failure modes of columns in G1.

The master column (C0) failed severely. This was followed by the buckling of the vertical reinforcement bars. The failure occurred when the concrete core reached its compressive strength limit. This caused major cracking, spalling of the concrete cover, and collapse due to crushing. As the concrete crushed and lost its ability to provide lateral support, the longitudinal reinforcement, particularly in compression zones, became unstable and underwent buckling due to insufficient confinement. The deficient column (DC) demonstrated buckling of vertical steel reinforcement bars at the ultimate stage, which is considered a critical failure mode in RC rectangular columns because the longitudinal reinforcement loses stability under high axial compression. At the ultimate load, concrete crushing and spalling reduced lateral confinement, leaving the reinforcement vulnerable to instability. This phenomenon is particularly pronounced in columns with insufficient steel reinforcement. The progressive nature of this failure significantly reduced the load-bearing capacity of the column, ultimately leading to structural collapse. This failure mechanism becomes more severe in columns with widely spaced transverse reinforcement, high axial load levels, increased slenderness, or lower reinforcement ratios. The failure modes for the reference columns (C0 and DC) are displayed in Fig. 8.

**Fig. 9:**
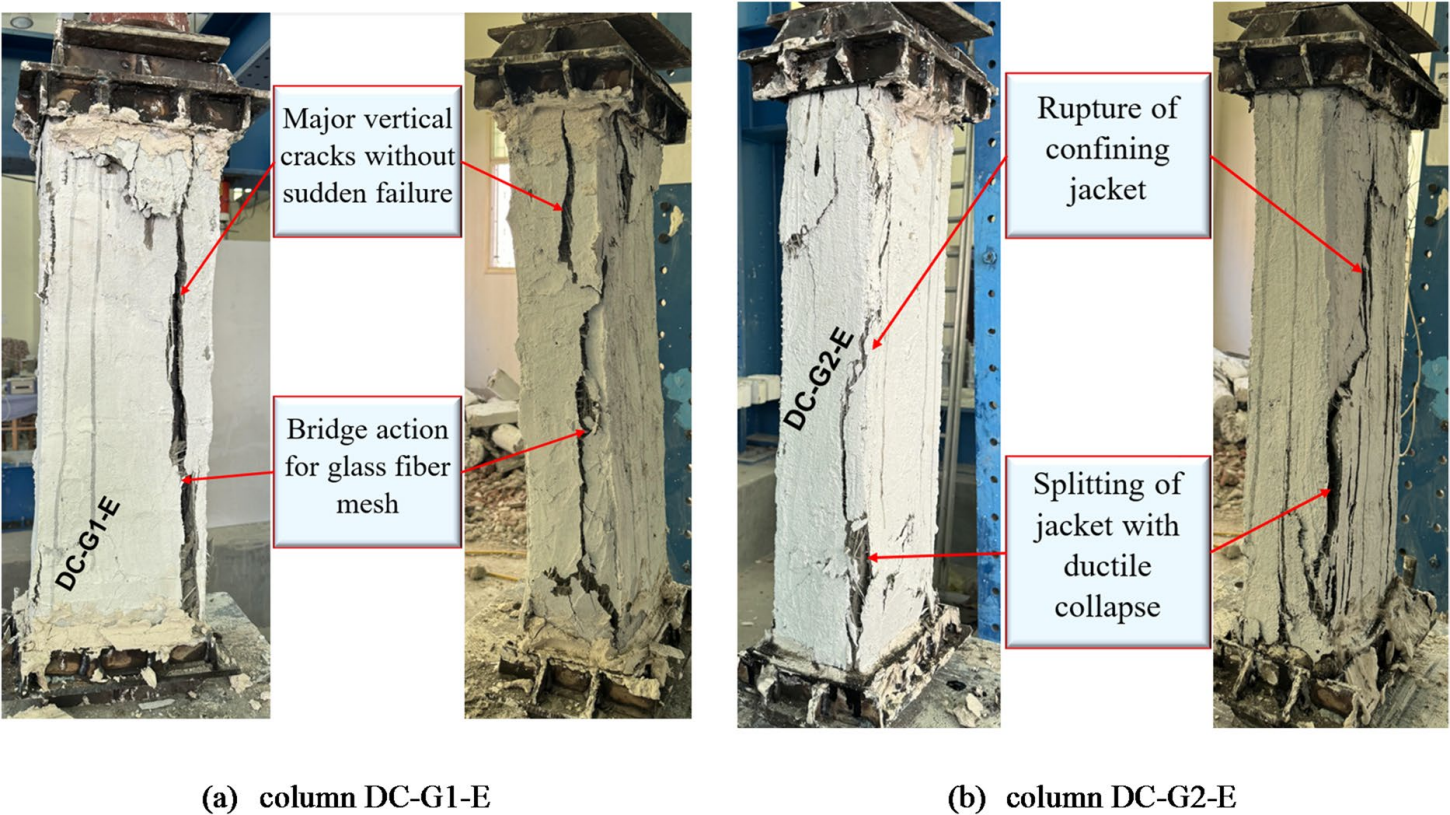
Failure modes of columns in G2.

For columns in group G2, column DC-G1-E, strengthened with a single layer of GFTM embedded in a GFTM-ECC, typically exhibited major vertical cracks without experiencing sudden failure due to the enhanced ductility and crack-bridging capability of the composite material. Unlike NC, ECC contains microfibers that improve tensile strain capacity, delay crack propagation, and prevent brittle failure. As axial load increased, vertical cracks developed progressively rather than leading to sudden crushing or splitting, allowing the column to sustain higher deformation before failure. Further, the embedded GFTM improved the load redistribution mechanism through a bridging effect in which the fibers transferred stresses across cracks, thus maintaining structural integrity even at advanced levels of damage. This bridge action improved post-peak behavior, delayed failure, and increased the overall ductility of the strengthened column. The failure in column DC-G2-E, which was reinforced with two layers of GFTM-ECC, was characterized by the rupture of the confining jacket and subsequent splitting within the jacket, leading to ductile failure. The GFTM-ECC jacket provided some initial confinement and thus improved strength and ductility; however, with an increase in axial load, the textile mesh approached its tensile capacity, causing rupture, which, in turn, resulted in a loss of confinement efficiency. The composite eventually lost integrity as the cracks grew through the jacket, enabling increasingly ductile deformation and a gradual loss of strength. These signs were characteristic of ductile failure. The failure modes for strengthened columns with a GFTM-ECC jacket are illustrated in Fig. 9.

**Fig. 10:**
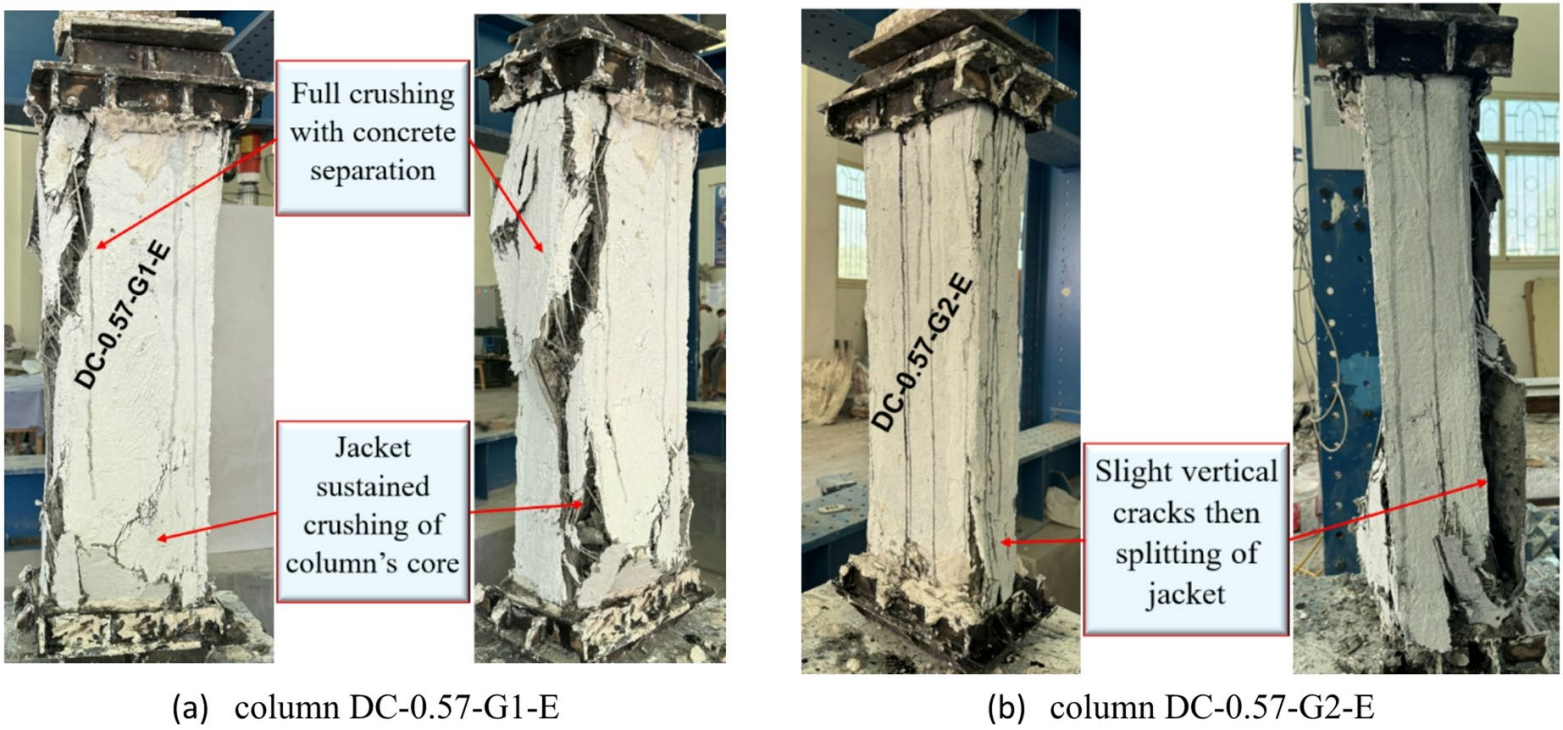
Failure modes of columns in G3.

In general, the utilization of NSM steel bars in upgrading rectangular RC columns improves their axial load-bearing capacity. Moreover, the application of an external jacket provides additional confinement and delays crack propagation. Column DC-0.57-G1-E, which was strengthened with four NSM steel bars covered with one layer of GFTM-ECC, demonstrated crushing and separation in the outer concrete. As the axial load increased, stresses in the concrete, steel, and jackets increased. Under high external stresses, the concrete experienced localized crushing, especially in regions of stress concentration. At this stage, separation between the original concrete surface and the strengthening layer of GFTM-ECC occured due to excessive strain growth and the initiation of bond strength degradation. Therefore, column DC-0.57-G1-E showed concrete crushing as a failure mode. However, the applied strengthening system succeeded in enabling the column to reach a higher load level, which is evident from the observed stress redistribution and bond performance. The failure mode of column DC-0.57-G2-E, which was strengthened with four NSM steel bars covered with two layers of GFTM-ECC, was characterized by the formation of slight vertical cracks followed by splitting in the external jacket. The additional confinement provided by the second layer of GFTM-ECC improved the structural integrity of the columns and prevented the development of severe damage. On the other hand, as loading increased, external vertical cracks developed, particularly in regions of stress concentration. With continued loading, deformation increased and cracks propagated, leading to splitting failure in the outer jacket. This failure mode shows that the strengthening system was successful in enhancing the column’s ductility and mitigating crack propagation, resulting in a higher axial load-bearing capacity. The failure modes for strengthened columns with NSM steel bars and GFTM-ECC jackets are represented in Fig. 10.

**Fig. 11:**
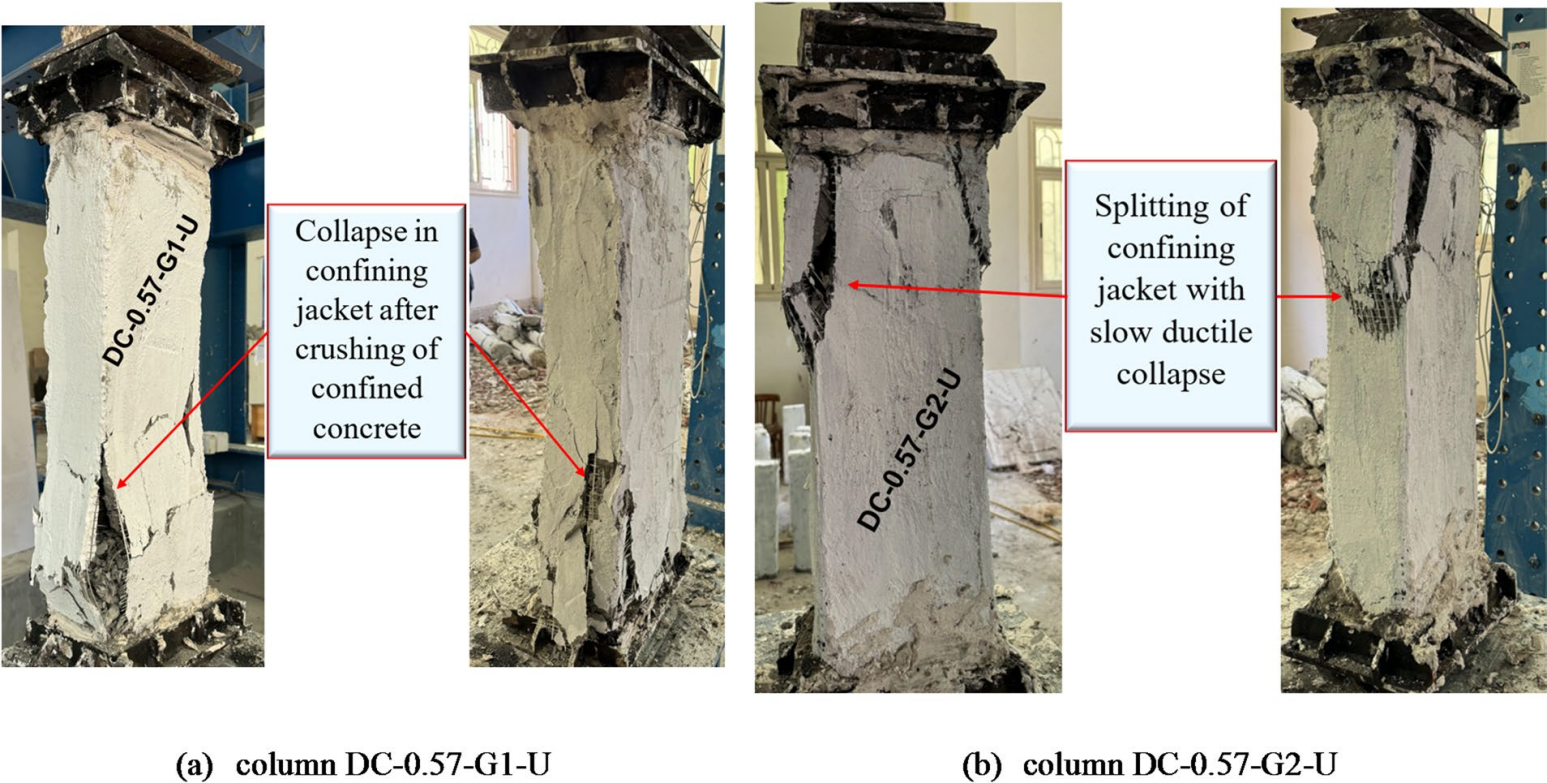
Failure modes of columns in G4.

The failure mode of column DC-0.57-G1-U, which was strengthened with four NSM steel bars and covered with one layer of GFTM-UHPC, is characterized by crushing of the concrete followed by collapse of the confining jacket. The application of the GFTM-UHPC layer provided superior confinement to the column and enhanced its axial load-bearing capacity. However, as the externally applied load increased, the confined concrete reached its crushing limit, leading to a sudden loss of the jacket’s integrity, which ultimately resulted in the jacket collapse due to the rapid release of internal stresses. In contrast, column DC-0.57-G2-U, which was strengthened with four NSM steel bars and covered with two layers of GFTM-UHPC, exhibited splitting in the confining jacket followed by a gradual ductile collapse. The additional GFTM-UHPC layer improved the confinement and delayed failure, allowing for more progressive deterioration. As the applied axial load increased, cracks formed in the jacket due to the development of the tensile stresses, leading to a slow ductile collapse instead of sudden failure. The provided strengthening system showed that the confinement and steel bars successfully enhanced the structural integrity of the strengthened columns, optimizing the bond between the concrete surface and the externally provided jacket. The failure modes for strengthened columns with NSM steel bars and GFTM-UHPC jackets are displayed in Fig. 11.

**Fig. 12:**
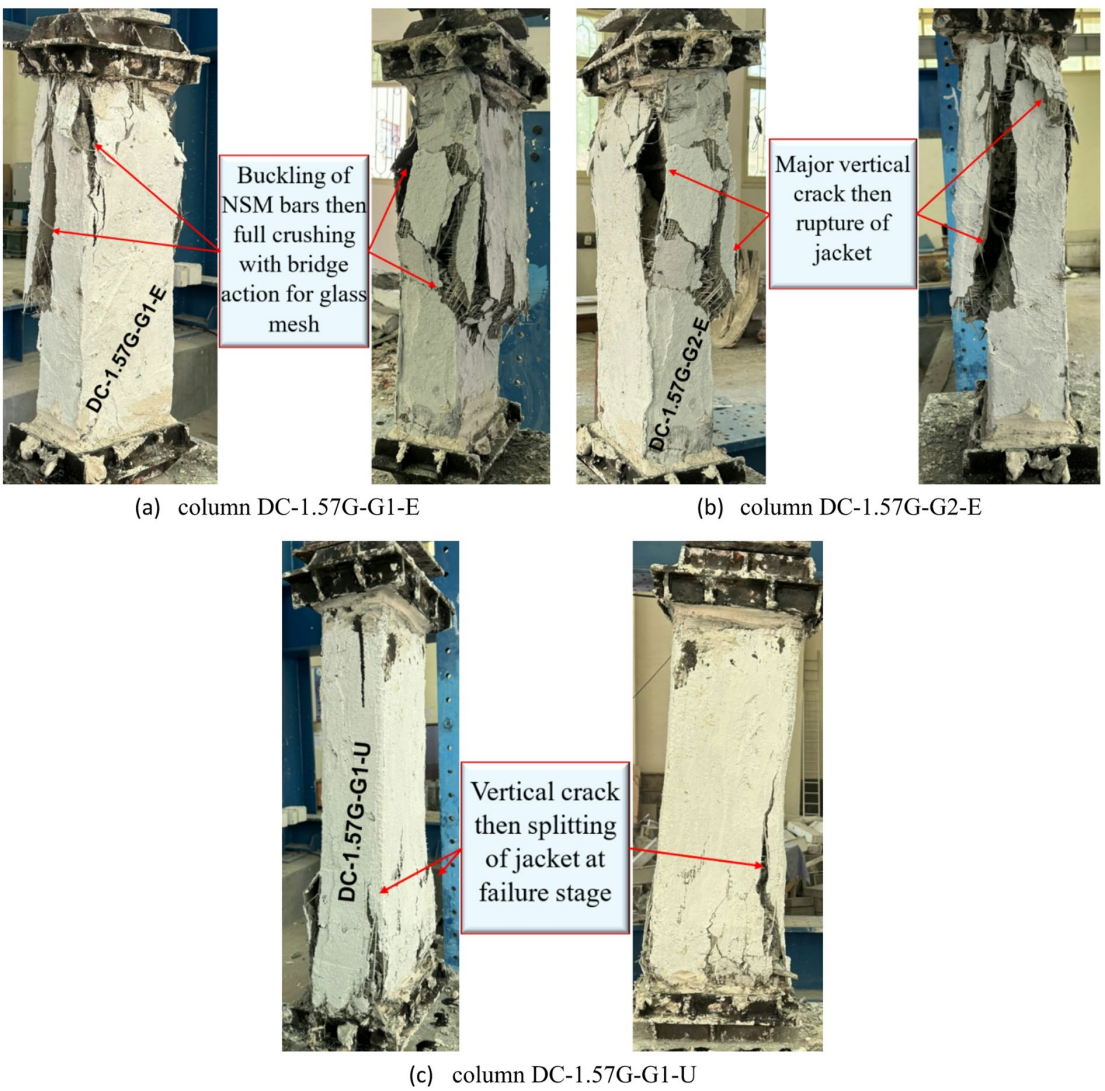
Failure modes of columns in G5.

Columns in group G5, strengthened with a hybrid system consisting of four NSM-GFRP bars and an external GFTM layer bonded by high-strength concrete, exhibit various failure behaviors depending on the number of GFTM layers and the type of strengthening concrete. The use of four NSM-GFRP bars and one layer of GFTM-ECC resulted in buckling failure of the GFRP bars, followed by full crushing of the concrete core. Moreover, as the load increased, GFTM temporarily bridges the load, delayed sudden failure, and eventually ruptured when the developed stresses exceeded its capacity. In contrast, the use of two layers of GFTM-ECC enhanced the structural performance of the RC column due to the additional confinement provided. Despite this improvement, failure still occured through the formation of vertical cracks. These cracks continued to propagate, ultimately leading to jacket rupture and a loss of confinement effectiveness. The column strengthened with four NSM-GFRP bars and one layer of UHPFRC showed improved initial confinement attributed to the superior mechanical properties of UHPC. However, at failure, vertical cracks developed in the column and expanded slowly as internal stresses were redistributed, resulting in jacket splitting. This indicates that premature cracking and splitting of the jacket can still occur, even with the enhanced strength offered by the UHPFRC jacket. The failure modes for columns in group G5 are presented in Fig. 12.

### Load–vertical displacement relations

Load–vertical displacement relationships for RC columns describe the structural response of columns under axial loading, illustrating how displacement values change as the applied load increases. These curves provide important information about column ductility, maximum load-bearing capacity, and failure mechanisms. By analyzing these curves, structural engineers can evaluate the efficacy of strengthening techniques, compare the results obtained from different column configurations, and optimize structural designs for enhanced resilience and safety.

**Fig. 13:**
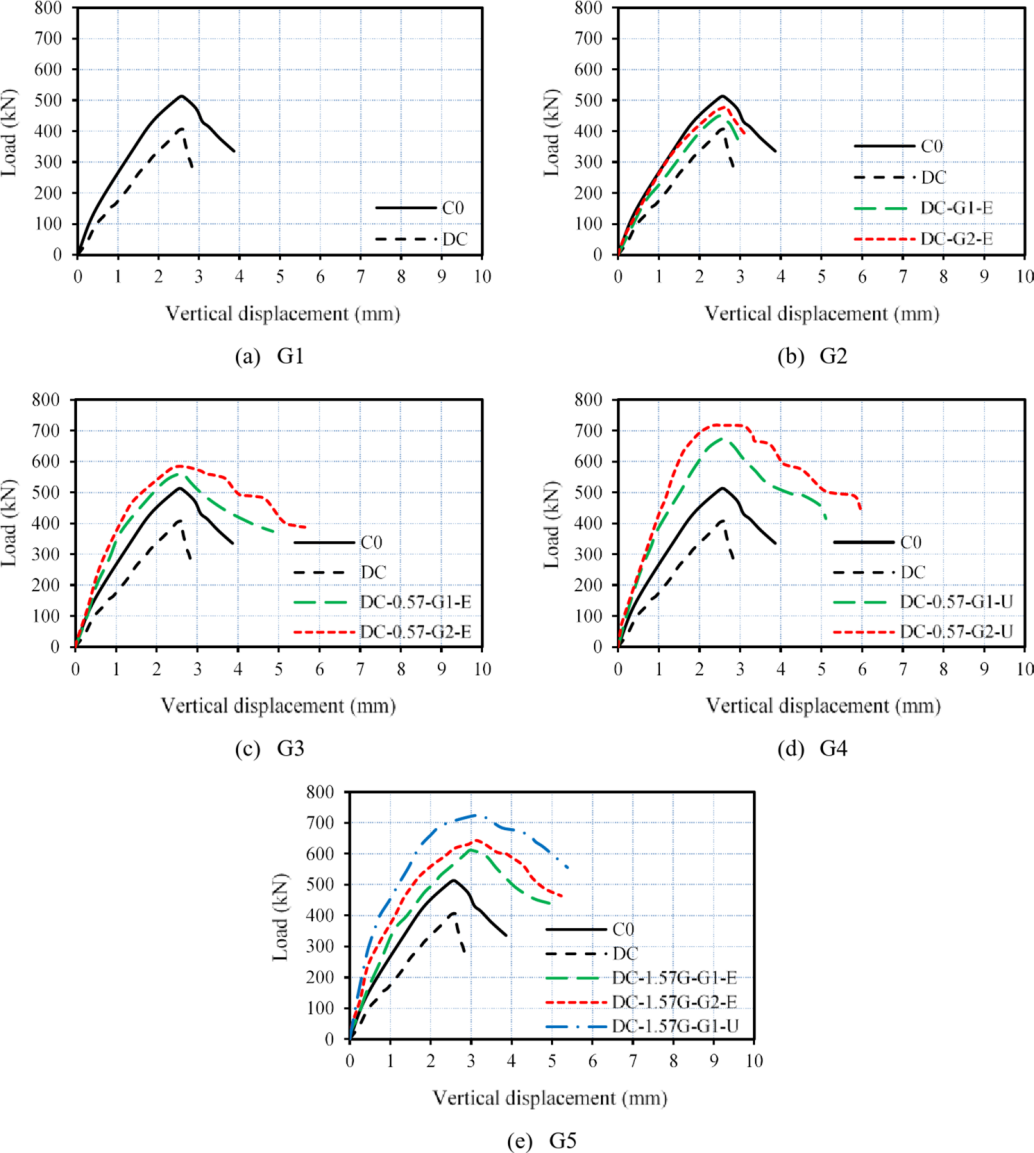
Load–vertical displacement relationships of tested columns.

The load–vertical displacement curves for all tested columns are displayed in Fig. 13. As shown in Fig. 13a, the deficient RC column exhibited lower axial load and lower ductility values when compared with those of the master column. Furthermore, Fig. 13b indicates that columns strengthened with only external GFTM-ECC layers demonstrated higher structural performance than the deficient column but did not reach that of the master column. On the other hand, Figs. 13c, d, and e reveal that strengthening deficient RC columns with a hybrid system outperformed the structural performance of the master column. In addition, this led to a notable improvement in their structural performance in terms of maximum axial load, ductility, and absorbed energy. As a result, strengthening RC columns by hybrid systems that combine NSM and external jacketing as confinement proved to be an efficient strategy. This system enhances the overall structural behavior of the columns.

**Fig. 14:**
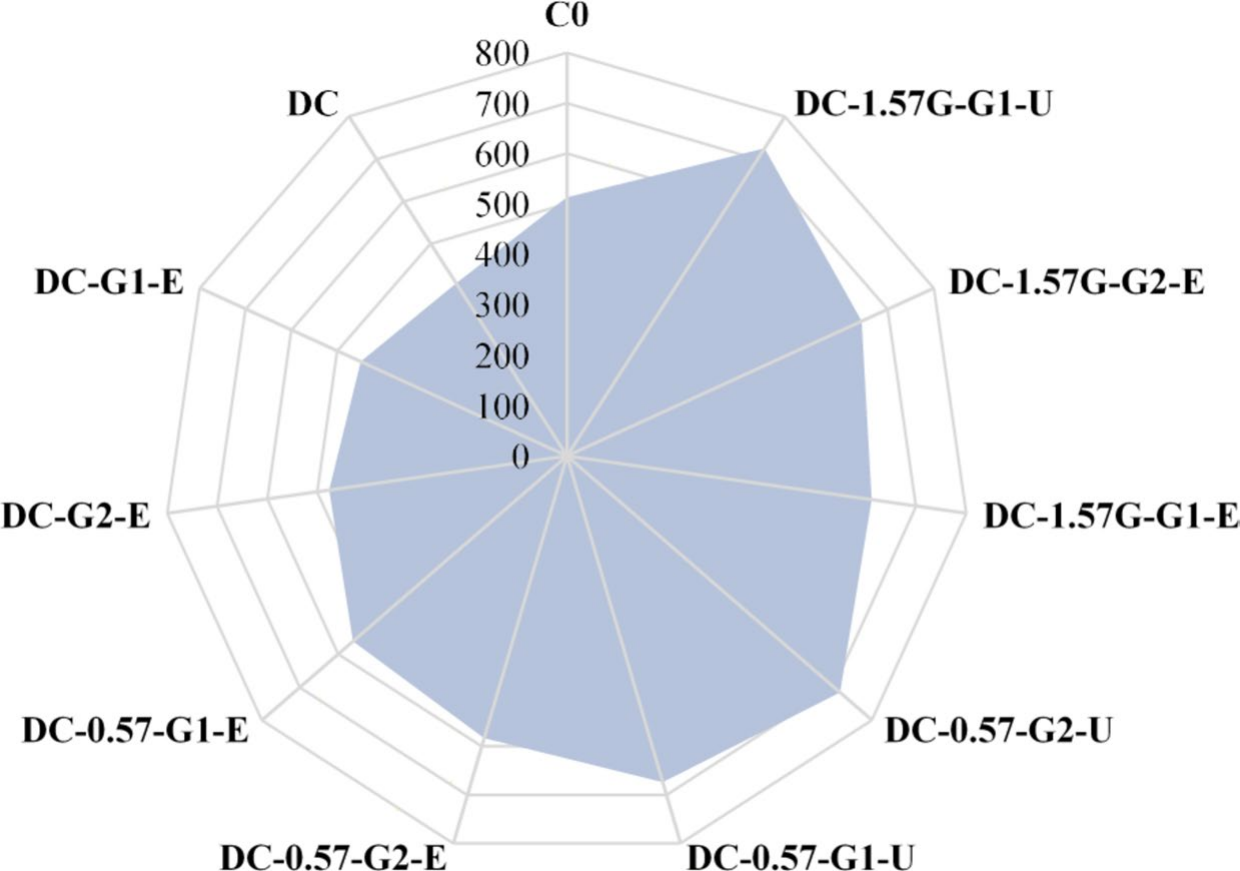
Comparison between ultimate loads for all tested columns (units: kN).

Table 4 summarizes all test results, highlighting the effectiveness of different strengthening techniques. Moreover, a comparison between the ultimate loads for all tested columns is depicted in Fig. 14. The test results demonstrate that an increase in the maximum axial load between 11% and 78% was achieved in the strengthened columns compared with the deficient column. The use of a hybrid strengthening strategy proved to be successful in restoring the axial load-bearing capacity of RC columns with insufficient reinforcement, as evidenced by the ultimate load values presented in Table 4. Notably, all columns strengthened with hybrid external reinforcement achieved a capacity higher than that of the master column, which was equal to 512 kN. In contrast, using only external jacketing slightly improved the axial load-bearing capacity of the deficient columns, with percentage increases of 10.6% and 17.2% for specimens strengthened with one and two layers of GFTM embedded in ECC mortar, respectively. Finally, the ultimate load results showed that strengthening columns with four NSM steel bars and two layers of UHPFRC, or with four NSM-GFRP bars and one layer of UHPFRC, provided the maximum enhancement in axial load-bearing capacity.

### Absorbed energy

**Fig. 15:**
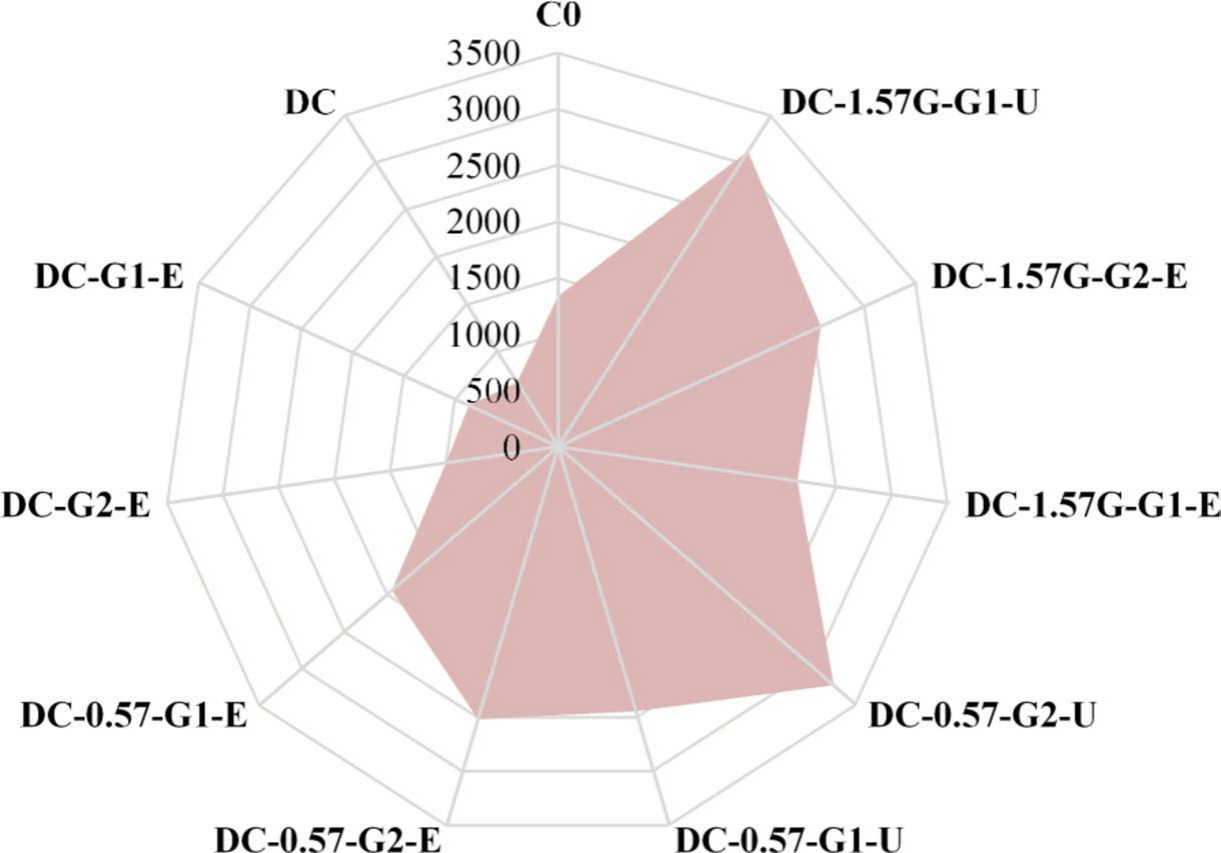
Comparison between absorbed energy for all tested columns (units: kN.mm).

The absorbed energy of RC columns represents the capacity of the columns to dissipate energy through material resistance and deformation before failure when subjected to external loads^26^. It reflects a column’s ability to withstand external applied loads, sustain deformations, and dissipate energy. In this study, the absorbed energy was calculated for each of the experimental columns to assess the effectiveness of the adopted strengthening techniques and to compare their performances. The absorbed energy results are listed in Table 4 and are visually compared in Fig. 15. The deficient column exhibited a considerable decrease in absorbed energy, to approximately 50% of that of the master column. This reduction was partially compensated by using external jacketing only and was fully compensated utilizing the hybrid strengthening system.

**Table 4:** Experimental results of tested columns.

Specimens’ ID	Ultimate stage				Absorbed energy (E)	E_C_/E_DC_	E_C_/E_C0_	Ductility index (µ)	µ_C_/µ_DC_
	P_u_ (kN)	P_uC_/P_uDC_	P_uC_/P_uC0_	Δ_Pu_ (mm)					
C0	512	1.26	1.00	2.59	1331	1.98	1.00	1.40	1.13
DC	406	1.00	0.79	2.62	671	1.00	0.50	1.24	1.00
DC	406	1.00	0.79	2.62	671	1.00	0.50	1.24	1.00
DC-G1-E	449	1.11	0.88	2.58	869	1.30	0.65	1.33	1.08
DC-G2-E	476	1.17	0.93	2.67	1003	1.49	0.75	1.41	1.14
DC	406	1.00	0.79	2.62	671	1.00	0.50	1.24	1.00
DC-0.57-G1-E	561	1.38	1.10	2.58	1948	2.90	1.46	1.45	1.17
DC-0.57-G2-E	583	1.44	1.14	2.48	2521	3.76	1.89	1.49	1.20
DC	406	1.00	0.79	2.62	671	1.00	0.50	1.24	1.00
DC-0.57-G1-U	674	1.66	1.32	2.56	2448	3.65	1.84	1.49	1.21
DC-0.57-G2-U	716	1.76	1.40	2.53	3233	4.82	2.43	1.51	1.22
DC	406	1.00	0.79	2.62	671	1.00	0.50	1.24	1.00
DC-1.57G-G1-E	610	1.50	1.19	3.02	2143	3.19	1.61	1.66	1.34
DC-1.57G-G2-E	642	1.58	1.25	3.14	2562	3.82	1.92	1.75	1.42
DC-1.57G-G1-U	724	1.78	1.41	3.12	3113	4.64	2.34	1.89	1.53

P_u_: Ultimate load; Δ_Pu_: Vertical displacement recorded at P_u_; E: Absorbed energy; µ: Ductility index.

When compared with the deficient column, the amount of absorbed energy increased by 30% and 49%, respectively, when one and two GFTM-ECC layers were applied. Using four NSM steel bars with one and two layers of GFTM-ECC jacketing increased the columns’ absorbed energy by 190% and 276%, respectively. Furthermore, applying four NSM steel bars with one and two layers of UHPFRC jacketing improved the columns’ absorbed energy by 265% and 382%, respectively. Moreover, the energy absorption capacity of the columns strengthened with one and two layers of GFTM-ECC jacketing and four NSM-GFRP bars increased substantially by 219% and 282%, respectively. A column strengthened with four NSM-GFRP bars and a single layer of UHPFRC jacket performed better, with a 364% increase in absorbed energy. These findings illustrate the efficacy of hybrid strengthening systems in significantly increasing the energy absorption of RC columns with insufficient reinforcement, making them more robust under external loads.

### Ductility analysis

Ductility is a key performance indicator that reflects the ability of RC columns to undergo inelastic deformation before failure. It is particularly important for deficient columns, as enhanced ductility improves structural safety by allowing the redistribution of stresses and preventing sudden brittle collapse.

In this study, the ductility of the tested columns was evaluated using a displacement-based ductility index defined as the ratio between the ultimate displacement and the yield displacement. Since a distinct yield point was not clearly observed in axially loaded columns, the yield displacement was determined utilizing a bilinear approximation based on 75% of the ultimate load. The calculated ductility indices are presented in Table 4. The results indicate that the deficient column exhibited a ductility index of 1.24, while the control (master) column showed a higher value of 1.40, corresponding to an improvement of approximately 13%. The application of external GFTM-ECC jacketing resulted in moderate ductility enhancement, where the ductility index increased to 1.33 and 1.41 for one and two layers, representing improvements of about 7% and 14%, respectively, compared with the deficient column.

More significant improvements were observed in columns strengthened using hybrid systems. For specimens strengthened with NSM steel bars and ECC jackets, the ductility index increased to 1.45 and 1.49 for one and two layers of jacketing, corresponding to improvements of approximately 17% and 20%, respectively. Similarly, columns strengthened with NSM steel bars and UHPFRC jackets demonstrated ductility indices of 1.49 and 1.51, indicating enhancements of about 20% and 22%.

The highest ductility improvement was achieved in columns strengthened with NSM-GFRP bars combined with external jacketing. The ductility index reached 1.66, 1.75, and 1.89 for different configurations, corresponding to improvements of approximately 34%, 41%, and 53%, respectively, compared with the deficient column. These improvements can be attributed to the combined effect of internal and external reinforcement mechanisms. The NSM bars contributed to increased load-bearing capacity and delayed internal damage, while the external GFTM and high-strength concrete jackets provided effective confinement, limiting crack propagation and enhancing deformation capacity. Additionally, the use of ECC and UHPFRC materials improves tensile behavior and energy dissipation due to their fiber-bridging capabilities, which play a significant role in enhancing post-peak performance.

## Numerical simulation

An NLFEA using ABAQUS software^27^ was generated in this study in order to validate the experimental findings. The significance of NLFEA lies in deriving reliable estimations of structural behavior for RC columns with insufficient reinforcement, which were then strengthened utilizing external reinforcements. A well-elaborated procedure for carrying out NLFEA is described in the following sub-sections.

### Model description and details

**Fig. 16:**
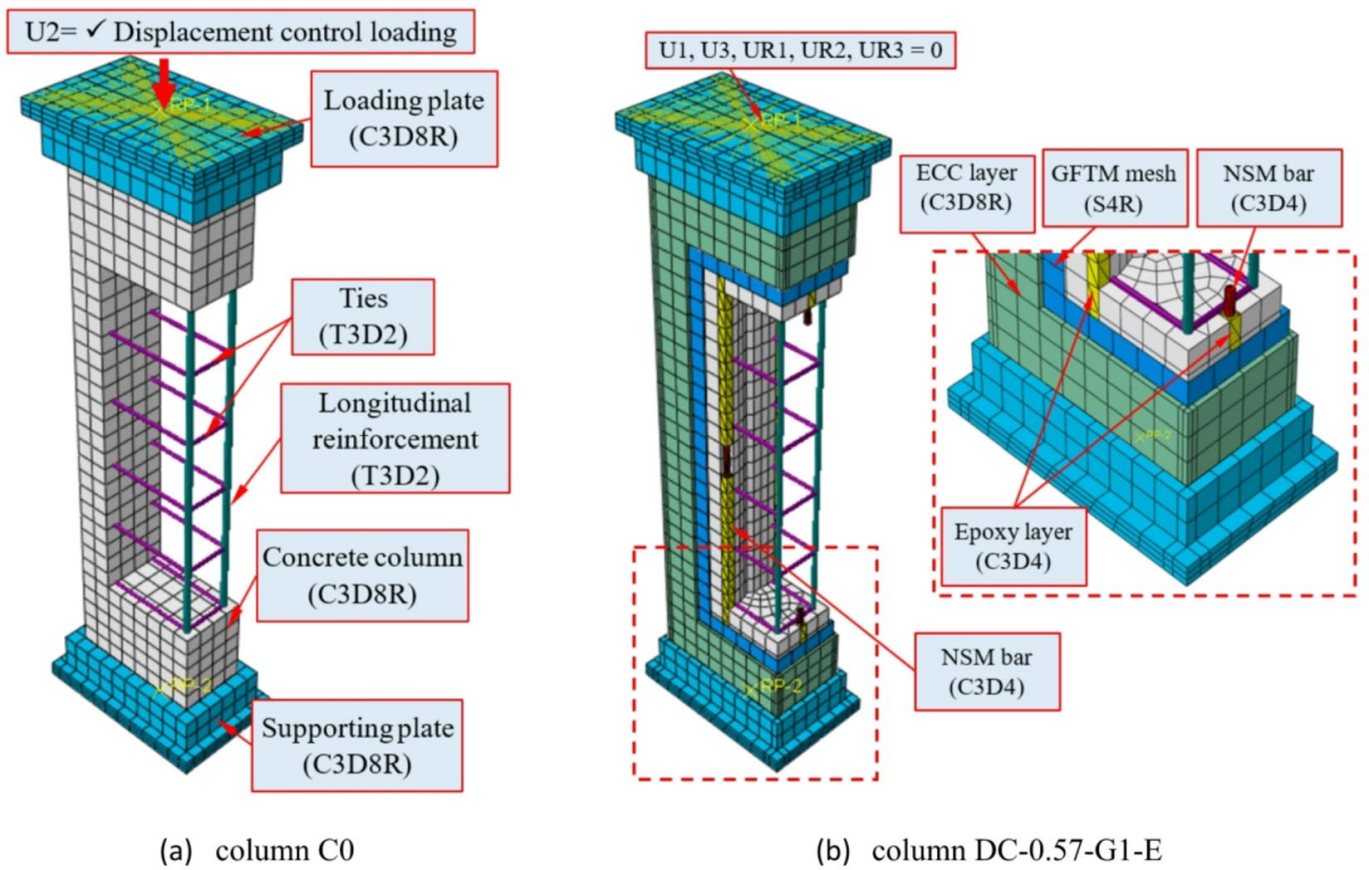
Numerical models of columns.

The concrete column, the supporting plate, the loading plate, and the high-strength concretes (i.e., ECC and UHPC) were modeled using the C3D8R element. The longitudinal reinforcing bars and transverse ties were simulated using 3D truss elements (T3D2). The GFTM was modeled utilizing the S4R element. Further, the NSM bar and the epoxy layer have been simulated employing the C3D4 element. An axial concentric load was applied at the column top using a displacement-controlled loading scenario. All created elements and a detailed description of the model components are illustrated in Fig. 16.

### Interaction between modeled elements

**Fig. 17:**
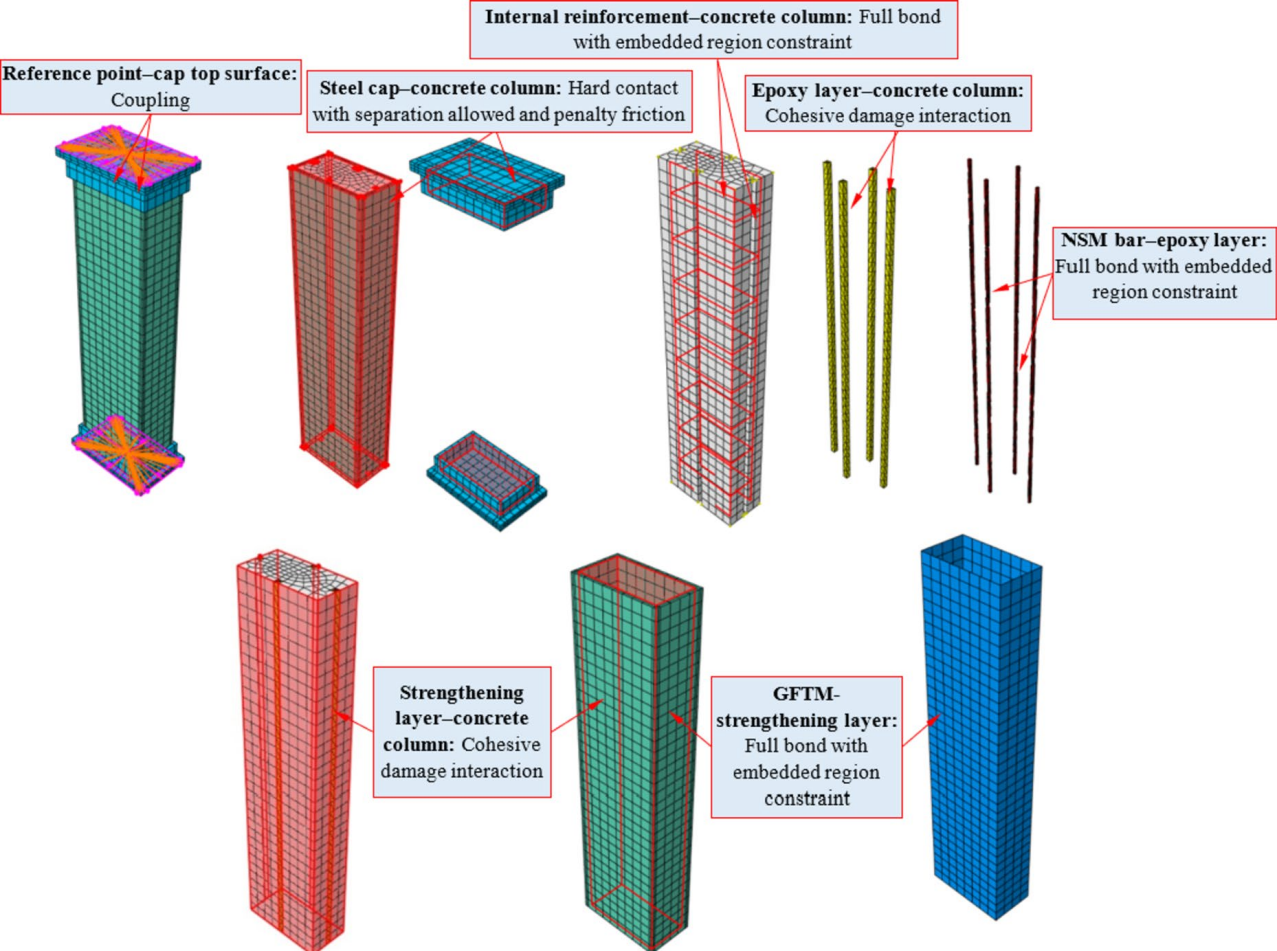
Interaction between different elements.

**Fig. 18:**
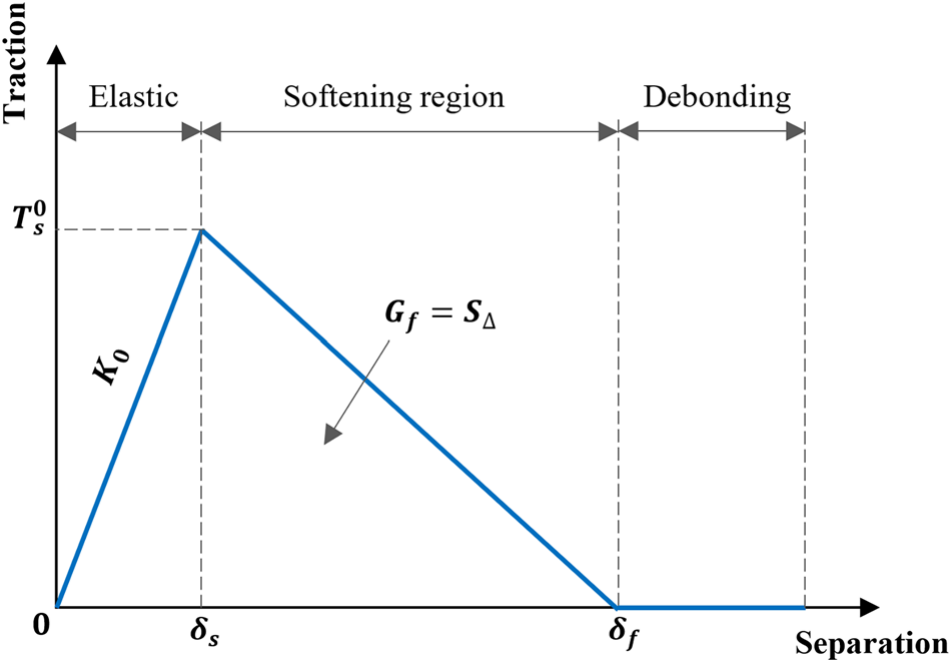
Cohesive damage interaction behavior^32^.

Element interactions in ABAQUS are very important, as they make it possible to mimic how different parts of a structure or assembly respond under different situations. These interactions include constraints, contact, and load transfer between parts or surfaces, all of which are provided in ABAQUS. Since element interactions are critical, they were implemented carefully and consistently for all components. The interaction between various elements in the RC column simulation is governed by distinct bonding and contact criteria to ensure effective load transmission and structural performance (Fig. 17). The internal reinforcement and concrete column maintained a strong bond through an embedded region constraint, which facilitated proper force distribution (i.e., the reinforcement served as the embedded region and the concrete served as the host region). The steel cap and concrete column interacted with hard contact, allowing for separation and frictional behavior, which enabled relative movement under loading. The epoxy layer and concrete column, along with the strengthening layer and concrete column, employed cohesive damage interaction, which permitted potential debonding and damage development. Figure 18 illustrates the behavior of the cohesive damage interaction. This contact progressed through three phases: the first phase represented the shear stiffness of the interaction; the second phase indicated the attainment of maximum shear stress at the interface; whereas the third phase demonstrated the post-peak behavior after reaching the maximum stress. The bonding of NSM bars with the epoxy layer was achieved through an embedded region constraint to ensure excellent stress transfer. Similarly, GFTM was fully bonded to the strengthening layer by means of an embedded region constraint, which maintained structural integrity^28^. Lastly, the reference point was connected to the top surface of the cap, ensuring an accurate representation of applied loads and boundary conditions. A schematic representation of the interaction between different elements created in ABAQUS software is displayed in Fig. 17.

A mesh sensitivity analysis was performed using mesh sizes of 15, 20, 25, and 30 mm to evaluate their influence on the predicted structural response. The results showed that reducing the mesh size to 15 mm led to a substantial increase in computational time with negligible improvement in accuracy, while the 30 mm mesh resulted in a slight reduction in prediction accuracy. The difference between 20 mm and 25 mm meshes was minimal; therefore, a mesh size of 25 mm was adopted as an optimal balance between computational efficiency and accuracy. The concrete column, supporting plate, loading plate, and high-strength concretes (ECC and UHPC) were modeled using the C3D8R element, which provided stable and reliable performance in capturing nonlinear behavior.

### Material modeling

The concrete damage plasticity (CDP) model was employed to simulate the nonlinear behavior of NC, ECC, and UHPC. The model parameters were defined based on literature recommendations and calibrated using experimental results^29–31^. The dilation angle was taken as 36° for NC, 34° for ECC, and 33° for UHPC, reflecting the plastic flow characteristics of each material. The eccentricity parameter was set to 0.1 to control the rate at which the flow potential function approaches its asymptote. The ratio of biaxial to uniaxial compressive strength (f_b0_/f_c0_) was assigned a value of 1.16, while the parameter K, representing the ratio of the second stress invariants, was taken as 0.667. Additionally, a viscosity parameter of 1 × 10⁻⁷ was adopted to enhance numerical convergence. These parameters enabled accurate simulation of damage evolution under both compression and tension, ensuring consistency between numerical predictions and experimental observations.

### Comparison of NLFEA and experimental results

When comparing NLFEA results with experimental results for axially loaded RC columns (for both reference and strengthened specimens), key parameters such as axial load-bearing capacity, maximum deflection, and failure modes should be evaluated. Typically, NLFEA provides details on stress distribution, crack patterns, and failure types. However, it is highly computationally intensive, highly dependent on input parameters, and requires an advanced level of expertise. Experimental testing delivers real-world accuracy, reflects the actual behavior of materials, and helps calibrate NLFEA models, but it can be expensive, limited in scope, and makes it difficult to conduct parametric analyses or assess internal responses. When used together, these approaches produce a synergistic effect, where experimental results validate and refine NLFEA models. Furthermore, this integration enhances overall understanding, enables effective parametric studies, and establishes reliable design standards. Both methods (i.e., experimental and NLFEA) should be integrated in the case of complex structural problems, which provides accurate and practical solutions.

**Fig. 19:**
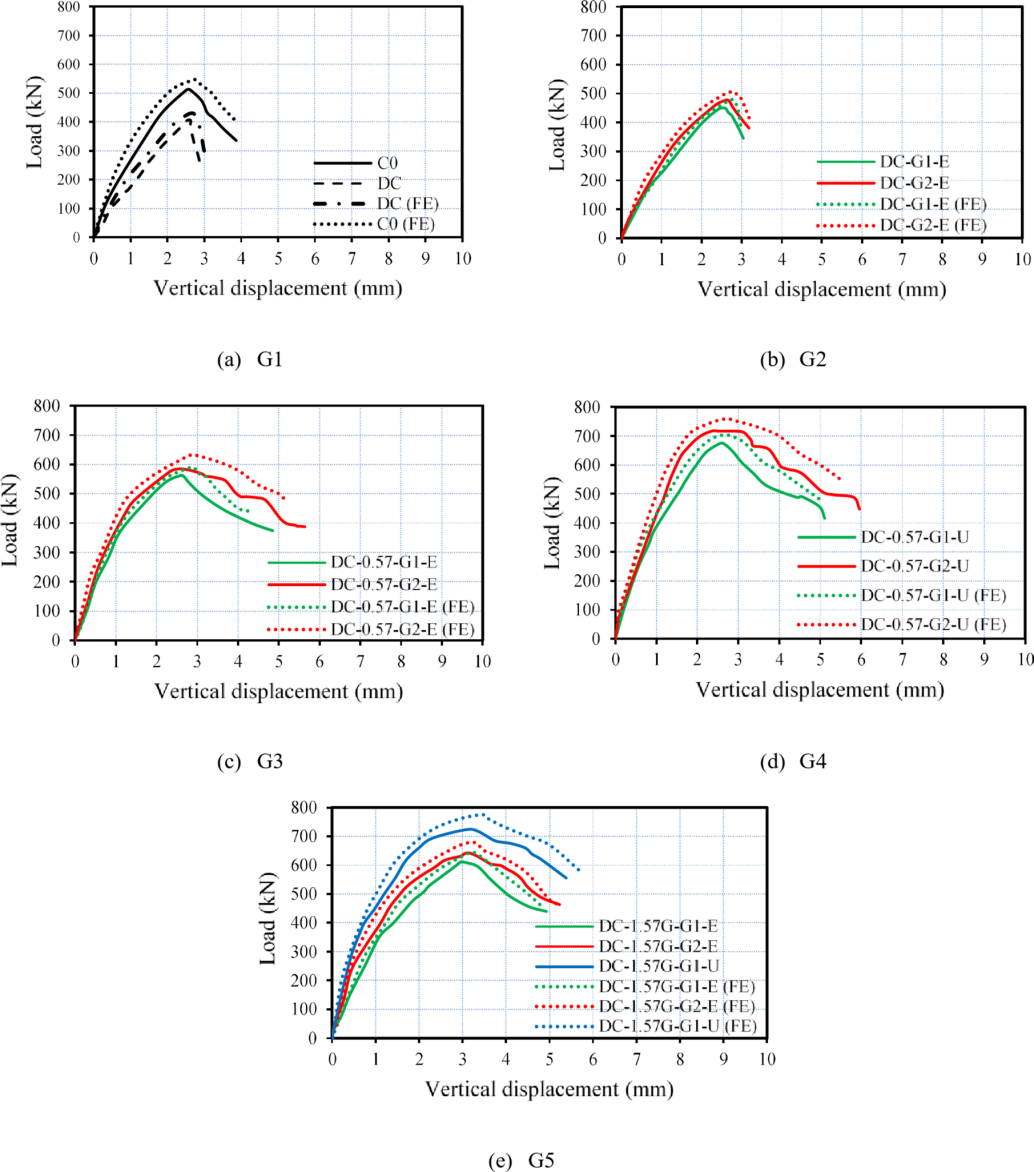
Comparison between experimental and numerical load–vertical displacement relations.

**Fig. 20:**
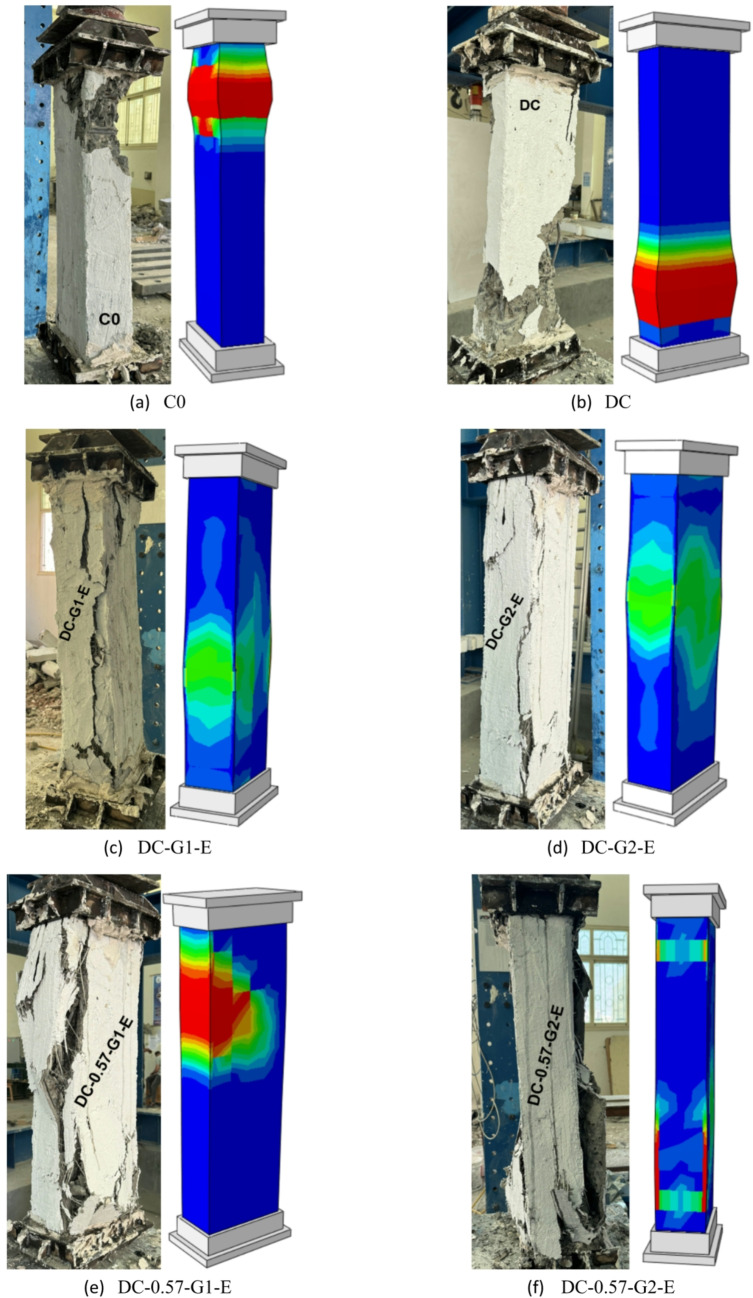

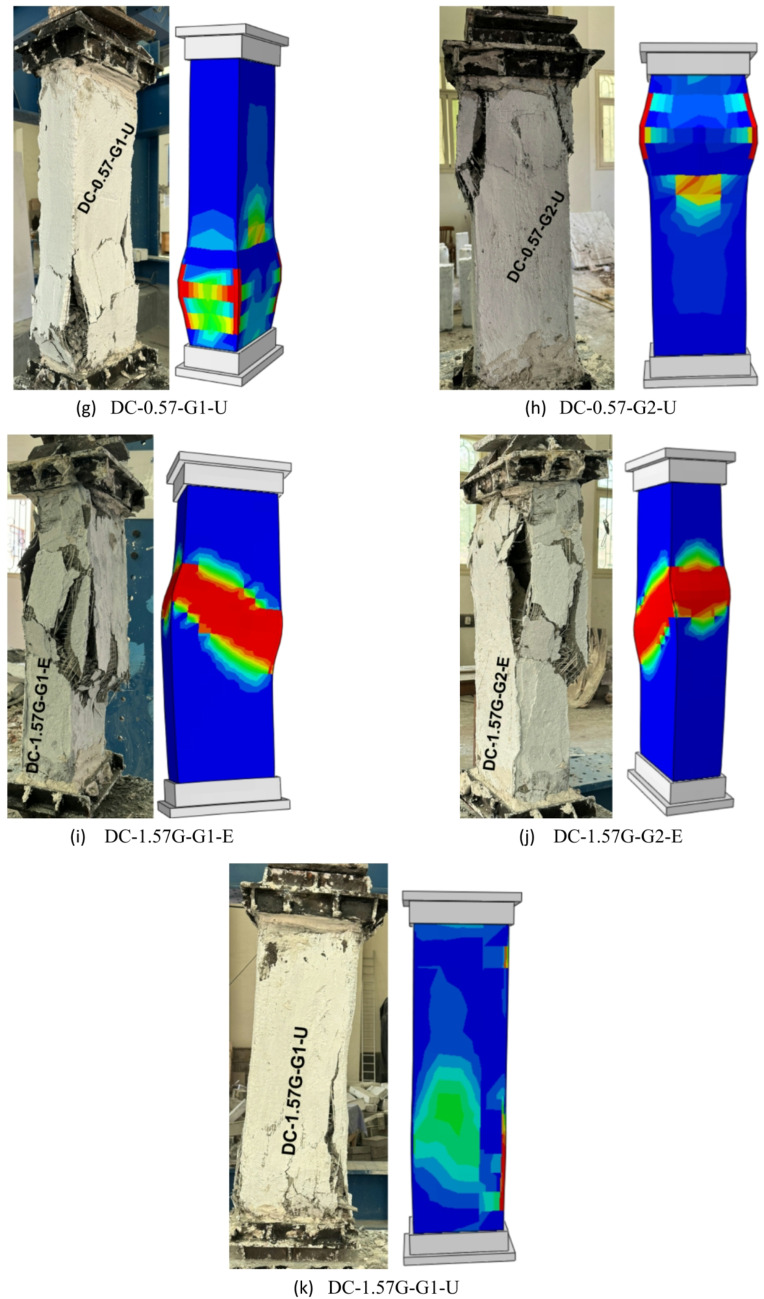
Comparison between experimental and numerical failure modes.

The current study developed NLFEA models using ABAQUS software to calibrate and validate the experimental results. Additionally, these models, in conjunction with experimental work, provided reliable predictions of the effectiveness of utilizing external jacketing or a hybrid system that combines NSM bars and external jacketing for strengthening deficient RC columns. Table 5 shows a comparison between the experimental and NLFEA results. Moreover, Figs. 19 and 20 illustrate the comparison of load–vertical displacement curves between the experimental and numerical results and their corresponding failure modes, respectively. The NLFEA models demonstrated a strong correlation with the experimental findings by accurately capturing the cracking patterns, failure modes, and load–vertical displacement responses. The data presented in Table 5 indicates that the differences between numerical and experimental results ranged from 4% to 8% for axial load-bearing capacity and from 3% to 14% for ultimate displacement, which validates the model’s accuracy in predicting the ultimate load-bearing capacity and displacement for both the control and strengthened columns.

During the development of the numerical model, several levels of vertical stress concentrations were intentionally applied to simulate the actual laboratory conditions observed during the experimental testing. These controlled stress distributions were adjusted to reproduce the real behavior of the tested columns, especially the localized failure zones and the progression of cracks under axial loading. This calibration process was essential to achieve strong agreement between the experimental and numerical results, particularly in predicting the failure modes and ultimate load-bearing capacities of the strengthened columns.

**Table 5:** Comparison between experimental and NLFEA results.

Specimens’ ID	P_u_ (kN)			Δ_Pu_ (mm)		
	EXP	FE	EXP/FE	EXP	FE	EXP/FE
C0	512	547	0.94	2.59	2.68	0.97
DC	406	422	0.96	2.62	2.73	0.96
DC-G1-E	449	478	0.94	2.58	2.78	0.93
DC-G2-E	476	505	0.94	2.67	2.81	0.95
DC-0.57-G1-E	561	589	0.95	2.58	2.89	0.89
DC-0.57-G2-E	583	633	0.92	2.48	2.87	0.86
DC-0.57-G1-U	674	703	0.96	2.56	2.65	0.97
DC-0.57-G2-U	716	759	0.94	2.53	2.68	0.94
DC-1.57G-G1-E	610	643	0.95	3.02	3.25	0.93
DC-1.57G-G2-E	642	678	0.95	3.14	3.27	0.96
DC-1.57G-G1-U	724	775	0.93	3.12	3.47	0.90
Avg			0.94			0.93
SD			0.01165771			0.0341058
CoV			0.00116577			0.0034105

EX: Experiment; FE: Finite element model; Avg: Average; SD: Standard deviation; CoV: Coefficient of variation.

To assess the accuracy of the numerical models, numerical and experimental results were compared by calculating deviations using standard deviation and coefficient of variation. The standard deviations for the axial load and the corresponding mid-height displacement were 0.01165771 and 0.0341058, respectively. The coefficients of variation for these parameters were 0.00116577 and 0.0034105, respectively. This comparison reveals that the model assembly, mesh size, boundary conditions, constraints, and material definitions are validated.

## Conclusions

This study presents an efficient strengthening system that aims to improve the performance of RC columns with insufficient reinforcement through the application of external reinforcement. The proposed strengthening methods include using external GFTM-ECC jackets or hybrid systems combining NSM bars (steel or GFRP) with high-strength concrete jackets using either ECC or UHPC. The study was conducted through experimental testing combined with NLFEA. From the results, the following main conclusions can be drawn:

- The structural performance of RC columns with insufficient reinforcement, strengthened using a hybrid system, exceeded that of the master column.
- An increase in the maximum axial load of strengthened columns in the range of 11% to 78% over the deficient column was recorded.
- Hybrid strengthening systems significantly enhanced the energy absorption capacity of RC columns with insufficient reinforcement.
- Energy absorption capacity demonstrated notable enhancement, with increases ranging from 30% for external jacketing to up to 382% for hybrid systems, indicating a substantial improvement in structural resilience.
- Ductility was also improved, where the ductility index increased from 1.24 for the deficient column to a maximum of 1.89, representing an enhancement of approximately 53%, particularly for hybrid systems incorporating NSM-GFRP bars.

## Data Availability

Data will be made available on request.
